# Properties and determinants of codon decoding time distributions

**DOI:** 10.1186/1471-2164-15-S6-S13

**Published:** 2014-10-17

**Authors:** Alexandra Dana, Tamir Tuller

**Affiliations:** 1 The Department of Biomedical Engineering, Tel-Aviv University, Tel-Aviv 69978, Israel

## Abstract

**Background:**

Codon decoding time is a fundamental property of mRNA translation believed to affect the abundance, function, and properties of proteins. Recently, a novel experimental technology--ribosome profiling--was developed to measure the density, and thus the speed, of ribosomes at codon resolution. Specifically, this method is based on next-generation sequencing, which theoretically can provide footprint counts that correspond to the probability of observing a ribosome in this position for each nucleotide in each transcript.

**Results:**

In this study, we report for the first time various novel properties of the distribution of codon footprint counts in five organisms, based on large-scale analysis of ribosomal profiling data. We show that codons have distinctive footprint count distributions. These tend to be preserved along the inner part of the ORF, but differ at the 5' and 3' ends of the ORF, suggesting that the translation-elongation stage actually includes three biophysical sub-steps. In addition, we study various basic properties of the codon footprint count distributions and show that some of them correlate with the abundance of the tRNA molecule types recognizing them.

**Conclusions:**

Our approach emphasizes the advantages of analyzing ribosome profiling and similar types of data via a comparative genomic codon-distribution-centric view. Thus, our methods can be used in future studies related to translation and even transcription elongation.

## Background

Translation elongation is an important stage of gene expression, known to affect the abundance, function, and properties of proteins and to have important contributions for the organism's fitness [1]. One fundamental question in the field relates to the way different features of the coding sequence and the intracellular environment affect the elongation dynamics and the properties of the encoded proteins. During the last decades, several studies aimed to answer this question, usually by correlating features of coding sequences with measurements of expression levels of endogenous and heterologous genes [2-10]. Among others, it was suggested that variables such as the adaptation of codons to the tRNA pool [2,3], codon order via their effect on tRNA recycling and ribosomal allocation [6,7], and the strength of mRNA folding in different parts of the transcript [9-12] contribute to the translation-elongation dynamics and protein abundance. Recently, it was demonstrated that codon-usage bias might also have a direct effect on various complex phenotypes and organismal fitness, such as circadian clocks [13-15].

Nowadays, the most promising experimental approach for studying the gene-translation process is the ribosome profiling method [16], which simultaneously enables estimating the relative time ribosomes spend on the mRNAs of all translated transcripts in a genome at nucleotide resolution. In this study, we have developed several computational and comparative methods to investigate several aspects of the codons' footprint count properties. These methods were applied on reconstructed ribosome profiles of thousands of genes, using previously published sequenced footprints of several organisms: *E. coli *[17], *B. subtilis *[17], *M. musculus *[18], *C. elegans *[19] and *S. cerevisiae *[20], significantly generalizing previous studies [17,21-23]. These techniques enabled us to show for the first time that each codon has its own characteristic footprint count distribution that tends to be preserved along the inner parts of the ORF but varies towards its ends. We discuss the codons' expected footprints distributions and compare them to their empirically measured distributions. In addition, we show that codon distributions tend to be more similar in the same domains of life.

Finally, we suggested several basic features of the footprint distributions and show that some correlate to the various proxies of intracellular concentrations of the tRNA molecules recognizing them. Our new suggested approach could pave the way to improved analyses of ribosomal profiling data and better understanding of the translation elongation dynamics and evolution.

## Results

In this study, we investigated several aspects of codon decoding time by reconstructing and analyzing the ribosome profiles of thousands of ORFs using previously published sequenced ribosomal-protected footprints of several organisms. The general aim of this study is to understand various aspects of the distribution of codon footprint counts, including: 1) its shape, 2) the way it varies along the ORF, 3) the way it varies between major organismal groups (prokaryotes and eukaryotes), and 4) an analysis of the basic features of the footprints distribution.

First we described the normalization approach we performed on the data to enable comparison between ribosomal-protected footprint counts of different genes, which resulted in **N**ormalized **F**ootprint read **C**ounts (NFC). Then, using these data, we inferred the NFC distribution of each codon type, representing the distribution of the codon's decoding time. Next, we performed a mathematical fitting of the NFC distributions to various distribution functions to characterize them. In addition, we showed that each codon has its own typical and unique NFC distribution. Moreover, we compared the NFC distributions among the analyzed organisms and demonstrated that intra-domain organisms (prokaryotes *vs*. eukaryotes) tend to have more similar NFC distributions.

Next, we investigated whether the characteristics of NFC distributions are preserved along the ORF. We showed that NFC distributions tend to be conserved at the inner part of the ORF but vary at both of its ends. Then we suggested new basic features of the NFC distribution and studied their relationship to the codon's decoding time via a simulative analysis of the translation process.

Finally, we analyzed these basic NFC features based on experimental data and demonstrated that some of the features correlate with the abundance of tRNA species recognizing them.

### Computing codons' relative decoding time and their distributions

Ribosome profiling is a new, experimental method that detects the momentary positions of ribosomes along the transcripts at nucleotide resolution. Thus, it provides high-throughput quantitative measures of the translational status of the entire transcriptome. The ribosome profiling experiment includes the following major stages: 1) Cells are treated (e.g., with cycloheximide) to arrest translating ribosomes; then mRNA molecules not protected by ribosomes are digested (e.g. by RNASE1).2) The RNA fragments protected by ribosomes are isolated and processed for Illumina high-throughput sequencing, resulting in ribosome-protected footprint reads.

By using a computational method (see Supplementary Methods in Additional file 1: Reconstructing ORFs ribosomal profiles of the analyzed organisms), the obtained sequenced footprints can be mapped to the transcripts of the analyzed organism, creating for each its own specific ribosomal footprints read count (RC) profile. These RC profiles can be used to infer various biophysical properties related to the translation-elongation process.

**Figure 1:**
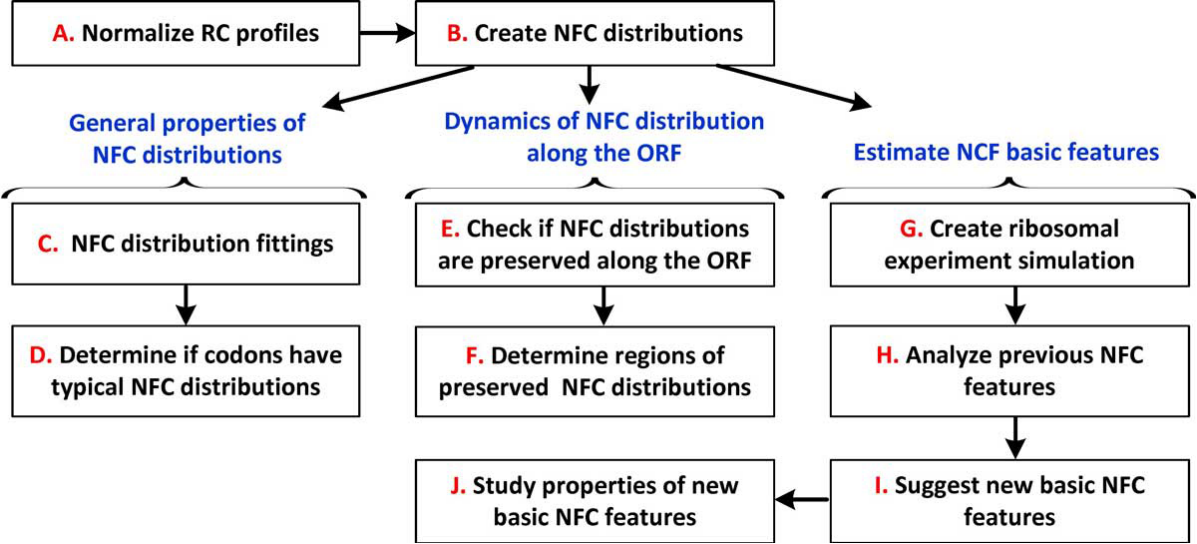
**Flowchart of the analysis steps performed in this study**.

Although slowly decoded codons create a higher amount of RC relatively to faster translated codons on the ORF, the absolute RC number of each codon along an ORF is also influenced and proportional to the mRNA levels of the gene and its translation initiation rate (see an illustration in Figure 01 in Additional file 1). Thus, to enable comparison of RCs measured from the different expressed genes of an organism, we normalized each RC profile by its mean RC, as was done in a previous study [17]. This normalization enables measuring the NFC of each codon in a specific ORF relative to other codons in it, while controlling for the two aforementioned factors that may influence the RC values of each codon (i.e., initiation rate and mRNA levels; see more details in Methods).

**Figure 2:**
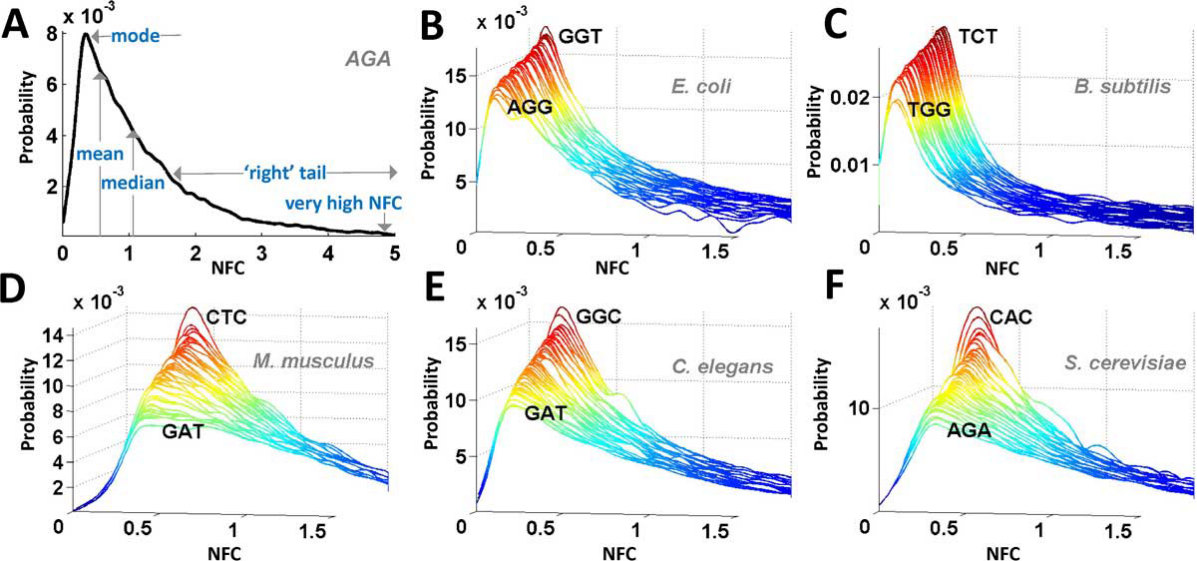
**NFC distributions**. (A) - Schematic description of the topology of the NFC distributions and some of its major features. The mode describes the NFC value that appears most frequently in the data. (B) - (F) - The NFC distributions of all codon types for the analysed organisms (after empirically fitting them to a curve), shown for NFC values the in range of 0 to 2. NFC distribution functions were sorted (front to back) according to the amplitude of their mode. The codon types with highest/lowest mode amplitude are marked in the figure.

To study the decoding-time properties of different codons, we generated a vector consisting of NFC values originating from all analyzed genes, for each codon type. We used these vectors to generate a histogram reflecting the probability of observing an NFC value of a codon in the expressed genes, for each codon type. Figures 2B-F and 08-12 in Additional file 1 show the NFC distribution of the various codons of the analyzed organisms in this study.

### Each codon has its own typical NFC distribution characteristics in both eukaryotes and prokaryotes

The first aim of the current study is to show that each codon has a distinctive NFC distribution (compared to other codons). As shown in Figures 2B-F, the general structure of the NFC distribution of different codons is relatively similar--it resembles a normal distribution but skewed towards NFCs with high values (the positive skew creates 'right tails' in the distribution; see example in Figure 2A); as we depicted below the shape of this distribution is usually close to log-normal.

**Figure 3:**
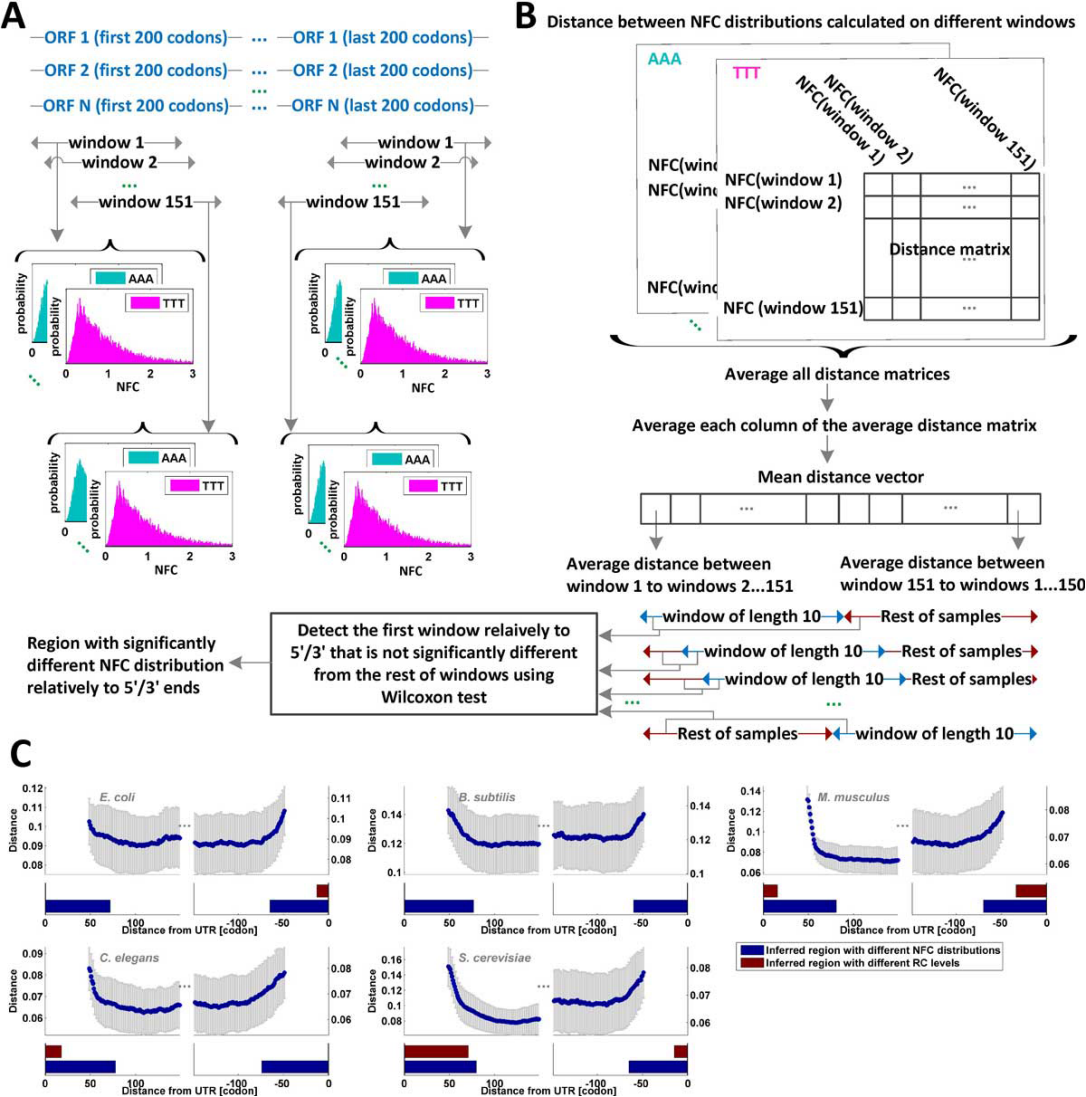
**Inferring the length of the regions at the ORF 5'/3' ends that are characterized by different NFC distributions relatively to inner parts of the ORF**. (A) - NFC distributions are calculated for each codon type, on windows of 50 codons, for the first and last 200 codons of the ORF. (B) Next, the distance between each pair of NFC distributions originating from different windows is calculated, creating a distance matrix for each codon type. The resulting distance matrices are averaged over all codons, and each column in the averaged matrix is averaged again, overall producing a mean distance vector. Each component in this vector describes the average distance between a NFC distribution calculated in the window it represents to NFC distributions of other windows (across all codon types). To determine at what location relative from the 5'/3' ends the distance between NFC distributions stop to significantly differ, a sliding window of length 10 was applied on the mean distance vector, and the values in and outside the window were compared using a Wilcoxon test. The first test that resulted in a p-value greater than 0.05 defined the location relative to the 5'/3' ends that was characterized by similar NFC distributions. (C) - Each subplot describes the mean distance vector calculated on the first and last 100 windows using the Hellinger metric (dotted graphs). The vertical bars depict the calculated standard deviation for each window. The navy bars beneath mark the regions relatively to the 5'/3' end with significantly different NFC distributions in comparison to subsequent regions on the ORFs. A similar test was directly applied on the averaged RC profiles (instead on the mean distance vector; see Figure 24 in Additional file 1), shown in burgundy bars (absent bars indicate of no such region). To emphasize the difference within each organism, different y-scales were used for each organism. For a comparison between organisms using the same y-scale see Figure 21 in Additional file 1.

To show that each codon indeed tends to have a unique characteristic NFC distribution, we performed a statistical test based on randomly partitioning the analyzed genes of each organism into two subsets of equal size (partition repeated 100 times). To test the robustness of the results, we employed three distribution distance measures that are based on different principles: Jensen-Shannon (JS) distance [24], Hellinger distance [25], and Energy distance [26] (see more details in Methods and Supplementary Methods in Additional file 1: Measuring the distance between NFC distribution functions.) Given two distributions, each of these measures returns a higher value when the distributions are less similar; specifically, when the two distributions are identical, the distance between them is zero.

In all the analyzed organisms, the distributions of each codon type tended to be more similar (self-distance) than they were to the distributions of other codon types (p < 0.01; more details in Table 03 in Additional file 1 and Supplementary Methods in Additional file 1: Different codons have characteristic NFC distribution functions), supporting the conjecture that the translation time is at least partially codon dependent. In addition, we showed that different codons coding for the same amino acid have unique NFC distributions (Table 03 in Additional file 1). This property is also shown for all codons with identical nucleotide composition (Table 03 in Additional file 1).

To characterize the distributions of the various codon types mathematically, we fitted them to 14 different common distribution functions that could theoretically attain such topology using the maximum likelihood criterion (see Supplementary Methods in Additional file 1: Different codons have characteristic NFC distribution functions). Figure 13 in Additional file 1 indicates that for the majority of codons in all organisms, the obtained NFC distributions could be best mathematically described using a log-normal distribution (or *very similar *distributions, see Table 07 in Additional file 1). This result was maintained also when calculating the distribution of codons in different parts of the ORF (sliding window of 50 codons)--the NFC distribution of codons in all windows tended to be similar to log-normal (Figures 14, 15, 16, 17, 18 in Additional file 1).

### The distribution of codon decoding time is conserved along the inner part of the coding sequence

Next, we aimed at studying whether the characteristics of the NFC distribution functions are location dependent or whether they are constant along the coding sequence. Answering this question would enable better understanding of the biophysical aspects of the translation process.

To this end, we calculated the NFC distribution of each codon in various regions along the ORFs (using a sliding window of 50 codons with step size of one codon) for the first and last 200 codons. For each pair of windows, the similarity between the NFC distributions was calculated, resulting in a distance matrix for each codon type. Then, we computed the average distance between the NFC distribution of a specific window to the other windows (averaged across all codons), resulting in a *mean distance vector *described in Figure 3C and 19, 20 in Additional file 1 (for a schematic diagram of the process, also see Figures 3A-B.) These results indicated that for all analyzed organisms, the distance between NFC distributions of windows near the 5'/3' and the rest of the windows was notably higher than it was between other windows located in the inner parts of the ORF, regardless of the distance metric used.

**Figure 4:**
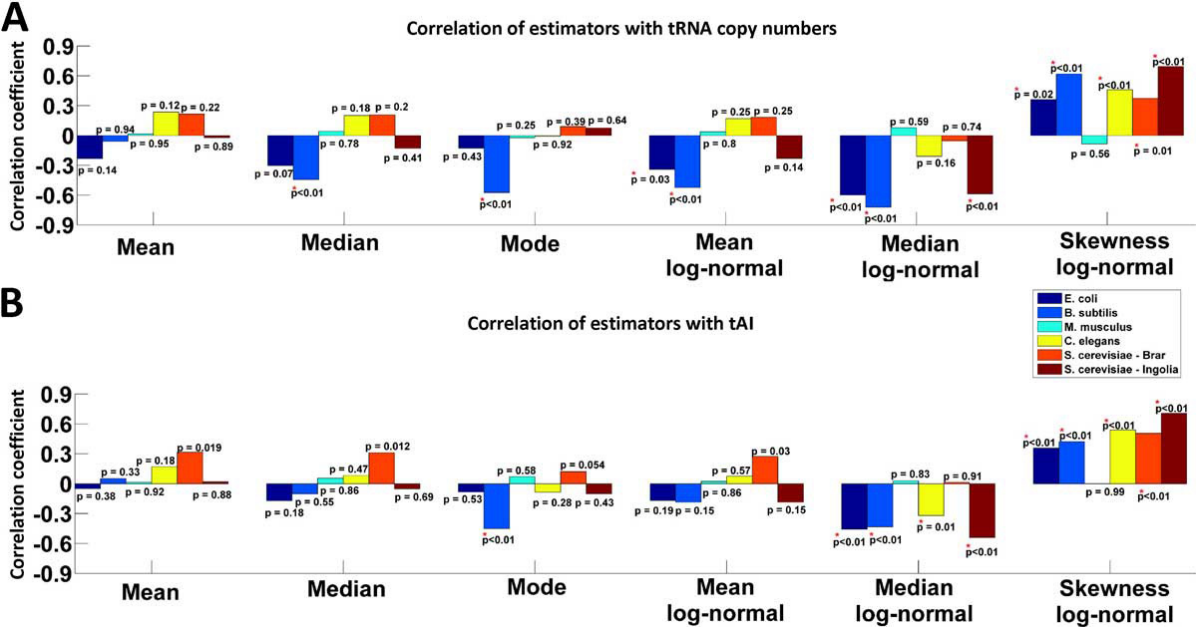
**The correlation of basic features of the NFC distribution with various proxies of tRNA levels**. Spearman correlations between various basic features (left to right: mean, median, mode, mean/median/skewness of the log-normal fitting) with tRNA copy numbers/tAI values for the different analysed organisms. The height of each bar is the Spearman correlation coefficient, significant correlations in the right direction (p < 0.05) are marked with red '*'.

To specifically estimate at what distance from the 5'/3' ends the NFC distributions start to become significantly similar to the rest, we compared each ten consecutive values to the rest of the values in the mean distance vector by using a Wilcoxon test. The first test that resulted in p-values greater than 0.05 defined the end of the region associated with significant different NFC distributions. The same analysis was applied to detect such a region relative to the 3' end (for a schematic diagram of the process, see Figure 3B).

For all utilized distance metric types, the region of significantly different NFC distributions was estimated to include the first ~76 codons and the last ~66 codons of the ORF (see also Table 04 in Additional file 1 and Figure 3C: blue bars). This suggests that NFC distributions of codons near the 5'/3' ends are significantly different from those calculated on the rest of the inner codons. The results were found to be robust to the length of the sliding window and the considered regions in the ORF (Table 05 in Additional file 1). This characteristic was also maintained when controlling for an equal amount of RC per window and codon type (as summarized in Table 06 in Additional file 1) to show that the reported results are not biased by the frequency of the codons in the ORFs [12,27-30]. This property was preserved also for the different gene ontology (GO) groups of *S. cerevisiae *(division by cellular component; see Supplementary Methods and Figures 22, 23 in Additional file 1).

For comparison purposes, a similar test was also applied on the average RC profiles of the analyzed organisms (Figure 24 in Additional file 1). This comparison showed that regions of different NFC distribution functions cannot be explained solely by the fact that in some organisms there is an observed increase of RC at the ends of the ORF [6,16] (See Figure 3C: burgundy bars).

## Comparing the NFC distribution among different organisms

To study the relationship between evolutionary distances among the various analyzed organisms based on the ribosomal profiling data, we calculated the distance between NFC distributions originating from different organisms for each codon type, resulting in a distance matrix. Next, we averaged the resulting distance matrix across all codons, and then clustered the organisms based on this distance matrix using the neighbor joining algorithm. The differences between the codon NFC distributions of various organisms indicated that codons of organisms from the same domain of life (prokaryotes vs. eukaryotes) tended to have more similar NFC distributions than did organisms from different domains of life (Hellinger distance: p = $2.7*10^{-16}$; see Figure 25 and Table 08 in Additional file 1).

## Basic features of the NFC distribution

Several translation-elongation factors can act as rate-limiting factors of the codon translation efficiency, such as the tRNA concentrations, aminoacyl-tRNA synthetase, and the binding time of the ternary complex of the ribosome. The combination of all these factors determines the total decoding time of each codon. Previous studies suggested averaging the NFC values of a codon to represent the codon NFC distribution, but found no correlation between them and tRNA levels in the cell [17,21-23]. In a previous study [31], we used the Totally Asymmetric Simple Exclusion Process (TASEP) simulation [32,12] to show that the mean NFC values (as calculated in previous studies [17,21]) are highly sensitive to phenomena such as translational pauses [17,33] and ribosomal jamming that result from codons with different decoding times (see additional details in the Supplementary Methods in Additional file 1).

Here we report additional basic features of the NFC distribution, showing in the next section that some of them correlate with proxies of the tRNA levels. As previously mentioned, in all the organisms analyzed in this study the log-normal function was found to best fit most of the codons' NFC distributions under the maximum likelihood criterion (Figures 2B-F and 08-12 in Additional file 1). This function could model the suggested positive skewness of the NFC values created by ribosomal jamming (see additional details in the Supplementary Methods in Additional file 1). Therefore, some of the suggested features were based on the log-normal function fitted to the NFC distribution.

Overall, we analyzed the following features of the NFC distribution: 1) the median; 2) the mode [34] of the NFC function, which describes the most frequent value in the data (see example in Figure 2A); and 3) statistical measures of the log-normal NFC distribution fitting, such as its mean/median/skewness (skewness measures the lack of symmetry in a distribution). For additional details regarding these measures see Methods.

Validation of these measures using the TASEP simulation (which included different decoding times for each codon type and translational pauses; see Supplementary Methods in Additional file 1) demonstrated that all these features highly correlated with the actual codon's decoding time. Specifically, the Spearman correlations for the different features were as follows: median: r = 0.7, ($p<2.3*10^{-10}$); mode: r = 0.96, ($p<3.3*10^{-65}$); mean of log-normal fitting: r = 0.58, ($p<1.2*10^{-5}$); median of log-normal fitting: r = 0.97, ($p<4.6*10^{-40}$); skewness of log-normal fitting: r = -0.95, ($p<2.3*10^{-30}$).

Interestingly, in this simulation the skewness of the log-likelihood fitting was found to correlate negatively with the simulated codon's decoding efficiency. This result indicates that, as expected, the NFC distributions of slower codons are less skewed, as they are less affected by delays caused by ribosomal jams and translational pauses.

## Correlation between basic features of the NFC distribution and measures of tRNA levels

Next, we calculated the newly suggested basic features based on experimental data. As the NFC distributions were previously shown to be preserved only in the inner part of the ORF, we calculated the codons' NFC distributions by using NFC values from the regions depicted in Table 04 in Additional file 1. Then we tested whether the suggested basic features related to the tRNA concentrations in the cell, as had been done in previous studies [17,21-23]. To this end, we used tRNA copy numbers and the tAI measure [3] as proxies for the tRNA levels [6,35]. For each of the suggested features, we calculated a Spearman correlation between the tRNA copy numbers and tAI measure, and summarized the results in Tables 11, 12 in Additional file 1 and Figure 4.

We found a significant correlation between the mean of the log-normal fitting and tRNA copy numbers for prokaryotes (-0.34 <r<-0.52; p < 0.033) and a significant correlation between the median of the log-normal distribution with tRNA copy numbers (-0.59<r<-0.72; p < 5.7*10^-05^) for the analyzed prokaryotes and yeast. The correlation between this estimator and the tAI values was found to be significant for the analyzed prokaryotes, yeast, and worm (-0.32<r<-0.54; p < 0.012). The correlation between the skewness of the log-normal fitting and tRNA copy numbers was also found to be significant in all organisms except for the mouse (0.36<r <0.69; p < 0.024); this estimator was also found to correlate with the tAI index (0.36 < r < 0.71; p < 0.0047). Further, this correlation remained significant when controlling for an equal amount of RC per codon type (Tables 13, 14 in Additional file 1), suggesting that this result is not biased by the appearance frequency of codons in the expressed genes.

However, the mode and median estimators also resulted in significant correlations to tRNA copy numbers and tAI values only for *B. subtilis *(Tables 11, 12, 13, 14 in Additional file 1), emphasizing the strength of the skewness of the log-normal fitting feature. To test the robustness of the features, we also calculated them on data of different experimental replicas. Spearman correlations between the suggested features (Table 15 in Additional file 1) were significant for all examined organisms, reinforcing their robustness.

Altogether, the detected negative correlation between proxies of tRNA concentrations in the cell and some basic features of the NFC distributions supports the conjecture that tRNA levels are one of the rate-limiting factors that affect codons' decoding time. In addition, these correlations could indicate a lower bound of the influence of the tRNA levels on decoding time, as the analyzed data could suffer from additional unknown noises and reduce the correlations. Furthermore, the tRNA copy number is clearly a proxy of the tRNA level, and it is very probable that the correlations with actual tRNA values are higher. By using the suggested features of the NFC distribution, the influence of additional factors that could act as rate-limiting on translation efficiency could be quantified in the future.

## Discussion

In this study, we analyzed for the first time the distributions of footprint counts in various organisms. We showed that in each organism, codons tend to have distinct NFC distributions. This emphasizes the importance of considering the entire NFC distribution range of codons, and not only their mean value, when studying various aspects of translation elongation.

One central result reported in this study relates to the fact that codons' NFC distributions tend to differ at the 5'/3' ends of the ORFs (compared to their inner parts). This result holds for all analyzed organisms and in *S. cerevisiae *for GO groups (cellular components ontology). Previous studies have suggested various signals related to translation control that are encoded at the beginning of the ORF [6,11,36-38], and that the translation-elongation speed is lower at the beginning of the ORFs [39]. However, here we suggest for the first time that the NFC distributions, and thus the elongation time, in the inner parts of the ORFs differ from their ends. This result suggests that for some aspects the translation-elongation stage can be refined by dividing it to three sub-stages: initiation-elongation, elongation, and elongation-termination. By measuring the change in the NFC distribution functions along the ORFs, we were able to estimate the length of these regions in various organisms and found them to be ~76 codons relative to the 5' end of the ORF and ~66 codons relative to the 3' end of the ORF. Several possible explanations could account for this observed phenomenon: 1) the translation elongation dynamic tends to change at the ends of the ORF due to changes in the nominal translation aspects (*e.g*. different conformation of the ribosomal structure at the ends); 2) the observed changes in the codons' translation-elongation distributions tend to change at the ORFs ends due to interactions with other macro-molecules (*e.g*., traffic jams at the beginning of the ORF [40,41], length of the peptide inside the exit tunnel of the ribosome) 3) we cannot exclude the possibility that the resulting signal is at least partially due to various experimental biases (*e.g*., [18,39]), although previous studies suggested that the effect of such possible biases is lower than the length of the regions reported in this study [18].

Straightforward analysis of the typical NFC values using direct estimators such as the mean used in previous studies [17,21] resulted in no significant correlation to the tRNA levels [17,21]. However, by analyzing basic features of the NFC distribution (*e.g*., the skewness of the log-normal NFC fitting), we were able to show that these values are correlated with tRNA levels. It is important to emphasize that the strength of the observed correlations between the NFC features and the proxies of tRNA levels reported in this study probably resulted from both biological phenomena and experimental biases. For example, the location of the P site of each fragment was determined according to the offset between the first peak of footprints and the initiation site, as had been done in previous studies [16,18,20]. However, the offset precision of the P site from the 5' ends of the fragments could vary along the ORF. Such variance could result from possible interactions between the ribosome and internal Shine-Dalgarno sequences in bacteria [42], the nuclease type that ribosomes used to remove unprotected fragments could cause a significant sequence bias because different nucleases usually digest (or digest more efficiently) only part of the nucleotides, or lysis buffer conditions, such as high magnesium concentrations, inhibit spontaneous conformational changes in bacterial ribosomes; therefore, reducing its concentrations could result in more complete and uniform nuclease digestion [20].

Further, different experimental protocols were employed to generate the data in the different studies used in this work; thus, we expected different levels of biases in the different analyzed organisms. The fact that most of the reported results are consistent in all/most organisms suggests that they are at least partially biological.

Based on ribosome profiling data, we provided basic features of the NFC distributions. Some of these features could be used for future studies in the field, replacing or adding to traditional measures of translation efficiency, such as tAI [3] and CAI [2]. Although the latter were vastly employed in previous studies [6,9,18,43-46], they are neither condition- nor tissue-specific, in contrast to the newly suggested features.

## Materials

### Calculating codons' normalized footprint RC--data normalization

To enable comparison and analysis of ribosome-protected RCs of codons of the same type originating from different genes, the RC of each codon was normalized by using the average RC in each gene. This approach was used also in a previous study [17].

Let us denote by  J the number of codons in the gene and  j is the index of a codon, then

$$NFC_{j}=\frac{RC_{j}}{\text{mean}\left(\text{RC}\right)}$$

As mRNA copies are expected to equally affect the RC of each codon on the gene, normalizing RCs by using the average RC of a gene cancels the effect that different mRNA levels have on codons originating from different genes. The RC values of codons are affected also by the initiation rate of ribosomes translating the mRNA: codons of genes with higher initiation rates will have higher values and *vice versa*. However, assuming this effect is also expressed in a relatively uniform increase in the RC along the gene, the normalization by the mean RC per gene also neutralizes it. Specifically, let us denote by $T_{j}$ the translation time of codon  j in gene  J and denote the mRNA levels of gene  J by  m and its initiation rate by  B. Therefore,

$$RC_{j}\propto m\cdot B\cdot T_{j}$$

and thus

$$NFC_{j}\propto \frac{m\cdot B\cdot T_{j}}{m\cdot B\cdot mean\left(T\right)}=\frac{T_{j}}{mean\left(T\right)}$$

This relationship indicates that NFC values represent the time a ribosome spends decoding each codon in a specific gene relative to the other codons in that gene. Hence, codons that are decoded faster will have lower NFC values than slower codons. Thus, regardless of the codon bias of a gene, slowly translated codons will tend to increase NFC values than will quickly decoded codons originating from the same gene.

Previous studies indicated an increase in RC at the beginning of the ORF [18,47] and, for some organisms, at the end of the ORF [17]. Therefore, for general presentation of the NFC distribution (e.g., when calculating the self-distance between NFC distributions of different codons or when mathematically fitting the general NFC distribution, as in Figure 13 in Additional file 1), the first and last 20 codons were excluded when calculating the average RC per ORF. We also excluded from the analysis codons containing less than one RC (as was done in a previous study [17]), to prevent biasing the average. Table 02 in Additional file 1 depicts the exact number of genes included in the analysis after applying this filter. We also evaluated the influence of the length of the ORFs on the NFC values and found the measure to be robust (more details in Supplementary Methods in Additional file 1).

### Reconstructing ORF ribosomal profiles of the analyzed organisms

The ribosome profiling reconstruction methodology was used as in a previous study [39,48]. Sources of the ribosome profiles and detailed reconstruction methods appear in the supplementary text in Additional file 1.

### Measuring the distance between NFC distribution functions

To test the robustness of the various results in this work, we chose to measure the distance between NFC distribution functions using three (out of dozens possible; *e.g*., see [49]) different distance metrics: 1) JS distance [24], 2) Hellinger distance [25], and 3) Energy distance [26], all of which are based on different concepts. The JS metric is based on the relative entropy, which was first defined by Kullback and Leiber [50] as a generalization of Shannon's entropy notion [51]. The Hellinger measure expresses the distance between distributions of vectors with independent components in terms of the component distances, whereas the Energy distance calculates the difference between two random variables (that create the NFC distributions), without specifically addressing their probability functions (For more technical details, see Supplementary Methods in Additional file 1).

### Mathematical fitting of the NFC distributions

The black histograms in Figures 8, 9, 10, 11, 12 in Additional file 1 depict the NFC distributions of each codon type for all analyzed organisms (*E. coli, B. subtilis, M. musculus, C. elegans*, and *S. cerevisiae*). The histograms were created by using the NFC values of all codons of the relevant type in the selected genes for analysis (except for the first and last 20 codons) relative to the 5'/3' ends of the ORF (see motivation in Methods: Calculating codons normalized footprint RC--data normalization). The codon's NFC distributions were fitted using various mathematical distribution functions that could describe natural processes: Beta, Birnbaum-Saunders, Extreme value, Generalized extreme value, Inverse Gaussian, Logistic, Log-logistic, Log-normal, Nakagami, Normal, Reyleigh, Rician, t location-scale. For each codon type and organism, the best fit was selected using the maximum likelihood criterion.

### Calculating tAI values of codons, tRNA copy numbers definition, and sources

The tRNA copy numbers are defined as the number of copies of each tRNA molecule that appears in the genome. The specific tRNA copy number of each codon and organism was downloaded from the tRNA genomic database [52]. The tAI index [3] describes each codon's adaptiveness to the tRNA pool. Additional details appear in the Supplementary Material in Additional file 1.

### Simulating ribosome density profiles using the TASEP model

Ribosome density profiles were simulated using the TASEP biophysical translation model, which is a stochastic model of ribosomal movement that considers the ribosome size, the different codon decoding times, the initiation rate, and possible interactions between ribosomes (traffic jams). Additional details about the TASEP simulation appear in the Supplementary Material in Additional file 1.

## Ranking the typical codon decoding times using statistical measures of the log-normal distribution

The log normal distribution is defined as

$$f\left(x\right)=\frac{1}{x\sigma \sqrt{2\pi }}e^{-\frac{{\left(lnx-\mu \right)}^{2}}{2\sigma^{2}}},x>0$$

The mean of the log normal distribution is defined as$e^{\mu +\frac{\sigma^{2}}{2}}$, its median is defined as $e^{\mu }$ and its skewness is defined as $\left(e^{\sigma^{2}}+2\right)\sqrt{\left(e^{\sigma^{2}}-1\right)}$[53].

## Conclusions

In this work, we studied novel properties of the distribution of codon decoding times by analyzing the ribosome profiling data of various organisms. The reported results demonstrate the advantages of analyzing various properties of codon NFC *distributions *rather than the (sometimes over-simplistic) trivial mean estimation of NFC values. In addition, we demonstrated the advantage of comparative analyses of these NFC distributions among organisms, genes, and different parts of the ORF. We believe that versions of the reported approach could be used in future studies related to translation elongation, codon bias, and transcript evolution. We also believe that the analyses performed in this work can be used in the future to study similar data related to other macromolecule movement in the cell (e.g., the movement of RNA polymerase during transcription).

## Competing interests

The authors declare that they have no competing interests.

## Authors' contributions

AD and TT analyzed the data and wrote the paper.

## Supplementary Material

Additional file 1 **This file contains description of 1) the method use for reconstructing genes ribosome profiles; 2) Method for evaluating the influence of length of the ORFs on the calculated NFC values; 3) Description of the applied measures for estimating the distance between NFC distribution functions;4) Description of method used for determining whether codons have characteristics NFC distribution functions; 5) Analysis details of NFC distribution properties for different GO functional groups; 6) Calculating codons' tAI values of codons; 7) Details regarding the profiling TASEP simulation**. This file also contains additional Figures and tables. Click here for file

## Declarations

This research is partially supported by Israel Cancer Research Fund (ICRF) and German-Israeli Foundation (GIF) I-2327-1131.13/2012. The remaining costs were funded by the authors' institutional resources.

This article has been published as part of *BMC Genomics *Volume 15 Supplement 6, 2014: Proceedings of the Twelfth Annual Research in Computational Molecular Biology (RECOMB) Satellite Workshop on Comparative Genomics. The full contents of the supplement are available online at http://www.biomedcentral.com/bmcgenomics/supplements/15/S6.
