# Supplementary material for: Properties and determinants of codon decoding time distributions
Source: BMC Genomics. 2014 Oct 17;15(Suppl 6):S13. doi: 10.1186/1471-2164-15-S6-S13 (PMC4240079; doi:10.1186/1471-2164-15-S6-S13)
Supplement: Additional file 1 — This file contains description of 1) the method use for reconstructing genes ribosome profiles; 2) Method for evaluating the influence of length of the ORFs on the calculated NFC values; 3) Description of the applied measures for estimating the distance between NFC distribution functions;4) Description of method used for determining whether codons have characteristics NFC distribution functions; 5) Analysis details of NFC distribution properties for different GO functional groups; 6) Calculating codons' tAI values of codons; 7) Details regarding the profiling TASEP simulation. This file also contains additional Figures and tables. [file 1471-2164-15-S6-S13-S1.pdf]

Properties and Determinants of Codon Decoding Time Distributions

Supplementary Material

Alexandra Dana and Tamir Tuller

The Department of Biomedical Engineering, Tel-Aviv University, Tel-Aviv 69978, Israel

Supplementary Methods

Reconstructing ORFs ribosomal profiles of the analyzed organisms

S. cerevisiae ribosomal profiles were reconstructed using the data published in the GEO database, accession number GSE34082 (GSM843780, GSM843781) [1]. Additional ribosome profiles were also processed from another *S. cerevisiae* experiment, accession number GSE13750 (GSM346111, GSM346114) [2]. *C. elegans* ribosomal profiles of genes expressed in the L4 larval stage were built from Illumina sequencing results (NCBI Sequence Read Archive, accession number SRR52883) [3]. *M. musculus* ribosomal profiles were reconstructed by using the data published in the GEO database, accession number GSE30839, sample ID GSM765292 [4]. *E. coli* and *B. subtilis* ribosomal profiles were built from the published Illumina sequencing results (GEO database, accession number GSE35641) [5].

Generally, prior to processing, the attached 3' linker or poly-A tail was removed from the fragments. To filter fragments originating from rRNA, those where first aligned to rRNA transcripts using the Bowtie software [6]. Fragments failing to align were further processed. To reconstruct the ribosomal profiles of the different genes, fragments were aligned to known exons and spliced junctions [2], allowing up to two mismatches.

The location of the ribosome's P site relative to the 5' end of each fragment was determined in a similar manner described in previous studies [2, 4] by calculating the offset between the 5' end of the most upstream mapped fragments to the initiation site. Previous studies showed that this offset slightly differs as function of the fragments length [2, 4], therefore in this study also the location of the P site relatively to the fragments 5' end was determined as function of the fragment's length. The location of the A site was therefore determined as the location of P site shifted downstream by three nucleotides.

Next, we have built a RC profile for each gene by increasing for each mapped fragment to the exon/spliced junction the counter of the nucleotides corresponding to the fragment's A site. Previous studies that reconstructed ribosome profiles discarded fragments that could be located

to multiple locations [4] [1] [7] [8]. A previous study [9] allowed mapping multiplicity. In this study we utilized all mapped fragments. Specifically, to overcome multiple mappings of a single fragment to an exon/spliced junction, the ribosomal RC profiles were built in two steps: in the first iteration only fragments aligning to a single location were mapped. For each successfully mapped fragment, the RC of the relevant A site (3 nucleotides) were increased by one. In the second iteration, for all fragments aligning to more than one location, the mean RC in the surrounding of the possible aligning locations was calculated (10 nt before and after the location of the A site, using only RC obtained from the first iteration); let RCM_i denote the mean RC in the surrounding candidate location i; a fragment was mapped to one of these locations, with the probability of

$$\frac{RCM_i}{\sum_i RCM_i}$$

where i depicts the index of a possible mapping location. The RC of relevant exons and spliced junctions were united to create for each gene transcript its ribosomal profile. Ribosomal RC profiles of codon resolution were calculated by averaging the RC of each three non-overlapping consecutive nucleotides. To increase the robustness of the data, for *S. cerevisiae*, *E. coli* and *B. subtilis* RC profiles of genes from different replications of the experiment were averaged together.

S. cerevisiae ribosome protected fragments were first removed from their poly A tail and fragments shorter than 20 bases were discarded [2]. Gene coordinates were taken from the Saccharomyces Genome Database - http://www.yeastgenome.org, reference genome S228c.

Fragments failing to align to rRNA transcripts were further processed and aligned to exons. The location of the most upstream mapped fragment was defined as the location of the first (upstream) read count peak, 10-25 nt before the beginning of the ORF (Figure 02, 03 in Additional file 1). Fragment lengths that failed to show a clear single peak were discarded. According to this analysis, for *S. cerevisiae* the location of the A site (offset from P site + 3 nt) was determined to start of 15-16 nt downstream of the 5' end of the fragment.

C. elegans footprints were removed from the 3' linker AGATCGGAAGAGCACACGTCTGAACTCCAGTCACCGTGATATC in the following manner: the 5' end of the linker was estimated to be located between nucleotides 20-36 of each processed fragment. Next, the Hamming distance between the estimated linker and the published linker was calculated (in terms of number of different nucleotides); a valid linker was accepted if this distance differed by up to two nucleotides. If no valid linker was found, the fragment was rejected [10]. Gene coordinates were downloaded from the UCSC genes data set [11], using the *C. elegans* WS220/ce10 genomic strain.

Fragments failing to align to rRNA transcripts were further processed and aligned to exons. Fragments that failed to show a clear single peak 10-25 nt before the beginning of the ORFs were discarded (Figure 04 in Additional file 1). These results indicate that the A site of *C. elegans* is located 14-15 nt from the 5' of the fragments.

M. musculus fragments were removed from the their 3' linker CTGTAGGCACCATCAATTCGTATGCCGTCTTCTGCTTGAA in a similar manner described for *C. elegans.* Gene coordinates were downloaded from the UCSC genes data set [11], using the mm9 genomic strain. Fragments failing to align to rRNA transcripts were further processed and aligned to exons. Those that failed to show a clear single peak 10-25 nt before the beginning of the ORFs were discarded (Figure 05 in Additional file 1). According to the results of this analysis, the A site of *M. musculus* was set 15/16/17 nt downstream for fragments of length of 29-30/31-33/34-36 nt accordantly (similarly to the results reported in the original study [4]).

E. coli and ***B. subtilis*** fragments were first removed from their attached 3' linker CTGTAGGCACCATCAAT, allowing up to one difference between the estimated linker and the real linker (due to its shorter length). The first nt of each fragment was ignored, as it frequently represents an un-templated addition during reverse transcription [12] and fragments shorter than 20 nt were discarded. Gene coordinates were downloaded from the Biomart database (www.biomart.org). Fragments failing to align to rRNA transcripts were further processed and aligned to genes transcripts. Fragments that failed to show a clear single peak 10-25 nt before the beginning of the ORFs were discarded (Figures 06-07 in Additional file 1). The results of this analysis indicate that the A site of *E. coli* is located 16-24 nt from the 5' of the fragments while the A site of *B. subtilis* is located 19-25 nt relatively to the 5' of the fragments.

To increase the robustness of the data, for *S. cerevisiae, E. coli and B. subtilis* RC profiles of genes from different replications of the experiment were averaged together. To avoid analyzing genes with low RC, only genes with a median RC above one were included in the analysis. Full statistics of the alignment steps for each processed dataset appear in Table S01.

Evaluating the influence of length of the ORFs on the calculated NFC values

To test whether the length of the ORF has a potential effect on the NFC values, we tested the influence of this factor in a controlled environment (TASEP simulation with translational pauses, as presented in Figure 28 in Additional file 1). All simulated genes were divided into two groups according to their length (top and bottom 50%). For each group we randomly selected an equal amount of 1200 NFC values for each codon type. Spearman correlation between the various estimated features on both group of genes was found to be highly correlative, indicating the robustness of the measure to the length of the genes (mean: $r = 0.92$, $p = 4*10^{-6}$; median: $r = 0.94$, $p = 4*10^{-6}$; mode: $r = 0.86$, $p = 2.5*10^{-7}$; mean of log-normal fitting: $r = 0.95$, $p = 4*10^{-6}$; median of log-normal fitting: $r =0.99$, $p = 2.7*10^{-6}$; skewness of log-normal fitting: $r = 0.91$, $p = 3.9*10^{-6}$).

Measuring the distance between NFC distribution functions

Let P and Q denote two probability measures, where $P = (p_1, p_2 \dots p_k)$ and $Q = (q_1, q_2 \dots q_k)$, where

$$0 \le p_i \le 1, 0 \le q_i \le 1, \sum_{i=1}^{k} p_i = 1, \sum_{i=1}^{k} q_i = 1$$

The Jensen-Shannon distance is defined as

$$JSD(P \parallel Q) = \frac{1}{2} D_{KL}(P \parallel M) + \frac{1}{2} D_{KL}(Q \parallel M)$$

where $M = \frac{1}{2}(P + Q)$ and $D_{KL}(P \parallel Q)$ is the Kullback-Leibler divergence [13]:

$$D_{KL}(P \parallel Q) = -\sum\nolimits_x p(x) \log q(x) + \sum\nolimits_x p(x) \log p(x)$$

The Hellinger distance is defined as

$$H(P, Q) = \frac{1}{\sqrt{2}} \sqrt{\sum_{i=1}^{k} \left(\sqrt{p_i} - \sqrt{q_i}\,\right)^2}$$

If X and Y are independent random vectors in R^d with cumulative distribution F and G respectively, then the energy distance between the distributions F and G is defined to be

$$D_{energy}(F, G) = 2\mathbb{E} \parallel X - Y \parallel \; - \mathbb{E} \parallel X - X' \parallel - \mathbb{E} \parallel Y - Y' \parallel$$

where $\mathbb{E}$ is the expected value and $\parallel \cdot \parallel$ denotes the length of the vector, resulting in

$$D_{energy}(F, G) = \frac{1}{nm} \sum_{i=1}^{n} \sum_{j=1}^{m} |x_i - y_j| - \frac{1}{n^2} \sum_{i=1}^{n} \sum_{j=1}^{n} |x_i - x_j| \; - \frac{1}{m^2} \sum_{i=1}^{m} \sum_{j=1}^{m} |y_i - y_j|$$

where statistical samples from X and Y are $x_1, \dots x_n$ and $y_1, \dots y_n$.

Different codons have characteristic NFC distribution functions

Genes containing sufficient RC (see Table S02) were randomly divided into two subsets of equal size, and for each subset the NFC distribution function of each codon type was calculated. Then, for each codon type the distance between its NFC distribution functions from the two subsets (self-distance) was calculated using various distance metrics (JS/Hellinger/Energy). In addition, the distance between the NFC distribution of each codon from the first subset to the distribution of

other codons from the second subset was also calculated. These two measures were calculated for each organism for 100 different divisions of the genes into two subsets.

Then, for each codon type we calculated the number of times the self-distance was smaller than the median distance to other codon types. We mark this result by the indicator I(condition), which results in the value 1, if the condition is met. Then, the empiric p-value quantifying the chance of measuring codons with self-distance that are bigger than the distance to other codons is defined for all codons as

$$p\_value = 1 - median\left(\frac{I_{codon_j}\left(self\ distance_j < median(distance\ to\ other\ codons)\right)}{N}\right)$$

where j depicts the index of the codon, $j = 1..64$ (not including stop codons) and N depicts the number of divisions, in our case $N = 100$.

To test whether codons coding for the same amino acid have specific NFC distributions, we compared the self-distance of a codon to the distances to other codons coding for the same amino acid. Therefore the p_value for this test was defined as

$$p\_value = 1 - median\left(\frac{I_{codon_j}\left(self\ distance_j < median(distance\ to\ other\ codons\ of\ same\ AA)\right)}{N}\right)$$

Amino acids coded by single codons were excluded from the analysis.

Similarly, to test if codons with the same nucleotide content have specific NFC distributions, we compared the self-distance of a codon to the distances of other codons that had the same nucleotide content. Therefore the p_value for this test was defined as

$$p\_value = 1 - median\left(\frac{I_{codon_j}\left(self\ distance_j < median(distance\ to\ other\ codons\ with\ the\ same\ nt\ content)\right)}{N}\right)$$

Analyzing the NFC distributions of codons in different regions of the ORF for different GO functional (cellular component) groups

To test if NFC distribution functions are location dependent, regardless of the genes' different functions, *S. cerevisiae* genes were also divided according to their gene ontology association (cellular component) (downloaded from http://www.yeastgenome.org/). NFC distribution functions were calculated for all genes in a GO functional group that were at least 300 codons long, to enable analysis of non-overlapping regions of the first and last 150 codons (this threshold was lowered to allow more genes to participate due to smaller group sizes), similarly using a sliding window of 50 codons.

Calculating tAI value of codons

The tAI index [14] describes the adaptiveness of each codon to the tRNA pool. Let's mark the adaptiveness value of codon of type i with W_i. Let n_i be the number of tRNA isoacceptors recognizing codon i. Let $tCGN_{ij}$ be the copy number of the j-th anti-codon that recognizes the i-th codon, and let S_{ij} be the selective constraint of the codon-anti codon coupling efficiency. Then, the absolute adaptiveness value of a codon is defined by

$$W_i = \sum_{j=1}^{n_i} (1 - S_{ij}) tCGN_{ij}$$

Let us mark the relative adaptiveness value of codon i with w_i, by normalizing each W_i with the maximal W_i value among the 61 W_i values. The **c**odons 'nominal' **t**ranslation **t**ime cTT_i used for the TASEP simulations was defined as

$$cTT_i = \frac{1}{w_i}$$

where codons with low adaptiveness values to the tRNA pool will be more slowly translated.

Simulating ribosomal density profiles using the TASEP model

Ribosomal density profiles were simulated using an adapted version of the TASEP biophysical translation model, previously used in different studies [15, 16]. This model considers all major aspects of translation elongation and initiation, including: the elongation speed of each codon (which in this study is based on the adaptation of the codon to the tRNA pool); initiation rate; the size of the ribosomes; interaction between ribosomes such as traffic jams and ribosomes blocking each other; and the fact that the process is stochastic.

Generally, in this model, the mRNA was simulated using a lattice of N sites, where N represents the number of codons of the ORF. Each ribosome was defined to cover 11 codons and its A site was located at the sixth codon. During translation, any codon could be covered at a time by a single ribosome at most. In each step of the simulation, a single ribosome was allowed to attach itself to the lattice/advance to the next codon if the first/next six codons were not occupied. The time between initiation attempts was set to be exponentially distributed with a constant rate λ. Similarly, the time between jump attempts from site i to site $i+1$ was assumed to be exponentially distributed with rate λ_i.

The time between events (initiation or jumping between sites) is therefore exponentially distributed (minimum of exponentially distributed random variables) with rate:

$$\mu(n_i) = \lambda + \sum_{i=1}^{N} n_i \lambda_i$$

where i describes the site (codon) number on the lattice and $n_i = 1$ if codon i is being translated, otherwise n_i=0. Therefore the initiation probability is given by $\lambda/\mu(n_i)$ and the probability of a ribosome to jump from site i to site $i+1$ is given by $n_i\lambda_i/\mu(n_i)$ [17].

To simulate the ribosomal profiling experiment, the TASEP model was adapted in the following manner [10]: footprint fragments were defined as fragments of the mRNA that were covered by a ribosome in the simulation. For each gene the TASEP model was run several times, proportionally to the measured mRNA levels of the gene. Thus each simulated profile of a gene was created from ribosome protected fragments from different mRNA copies, similarly to the real ribosome profile experiment.

In this study *S. cerevisiae* genes that contained a sufficient amount of RC (see criterion in Table S02) and with a least 50 codons were simulated. Codons translation rate λ_i were set according to their adaptiveness to the tRNA pool (specific used values in the simulation are presented in Table S11).

$$\lambda_i = w_i$$

The initiation rate λ was set to be as the translation rate of the fastest codon (unless mentioned otherwise). To reach a steady-state distribution on the mRNA, the simulation of each mRNA copy was run until 200 ribosomes finished translating the gene. At this point of the simulation, codons covered by ribosomes were referred as ribosomal protected fragments. For each simulated gene the TASEP model was run for m times, where m represents the mRNA level of the gene. This parameter was set to be the maximal measured RC value of each real gene. The protected fragments of each one of the m simulations of a gene was used to create its ribosomal profile as previously described for *S. cerevisiae*.

In the first TASEP simulation [18] we set all codons to have equal translation efficiency, with a low initiation rate so traffic jams will be seldom created. The NFC distributions in Figure 26 in Additional file 1 demonstrates that indeed in this scenario the NFC distributions are normally distributed (under KS-test MEAN P VALUE = 0.27).

However, codons with low decoding times could be more affected by ribosomal jams, a phenomenon reflected in 'right' tails of their NFC distribution. In addition, codons with high decoding times and translational pauses would create higher NFC read counts, also expressed in extreme values of the NFC distribution. Therefore, we expect the NFC distributions of slower codons to be less skewed (*i.e.* more symmetrical), as they should theoretically be less affected by delays caused by these factors. Thus, it is possible that a mean estimator could be too sensitive and easily biased by the skewness of the NFC distribution.

Therefore we created a second TASEP simulation [18] in which codons were set different translation efficiency values, and indeed we observed a right skewness of the NFC distribution (Figure 27 in Additional file 1). This result reinforced our hypothesis regarding the effect of ribosomal jams on the skewness of the NFC distributions of the different codons. To estimate if indeed ribosomal jams could bias the mean and the other suggested estimators, we calculated Spearman correlation between the simulated decoding time values for each codon type and the mean of the NFC values, however the result was highly significant (r = 0.99, $p < 3.5 * 10^{-52}$). This suggests that averaging NFC values could be an adequate estimator for codon decoding times, if indeed the translation elongation process is generated by a TASEP-like process.

To demonstrate that even a very small number of translational pauses can have a major effect on the mean estimator, we've altered 4% of the codons to cause translational pauses [18]. To this end, codons of the real *S. cerevisiae* ribosomal profiles with NFC higher than three-fold of the mean NFC of the ORF (when excluding codons with zero read counts[5]) were defined as locations of translational pauses. The translation time of these codons was set to be proportional to the NFC of the measured pauses in the real profiles. The resulting NFC distributions are presented Figure 28 in Additional file 1. However, Spearman correlation between the simulated decoding times and estimated mean NFC values (as calculated in previous studies [5, 7]) deteriorated to r = -0.11 ($p < 0.4$). This result indicates that the mean NFC values are highly sensitive to possible outliers caused by the additional translational pauses.

Supplementary Figures

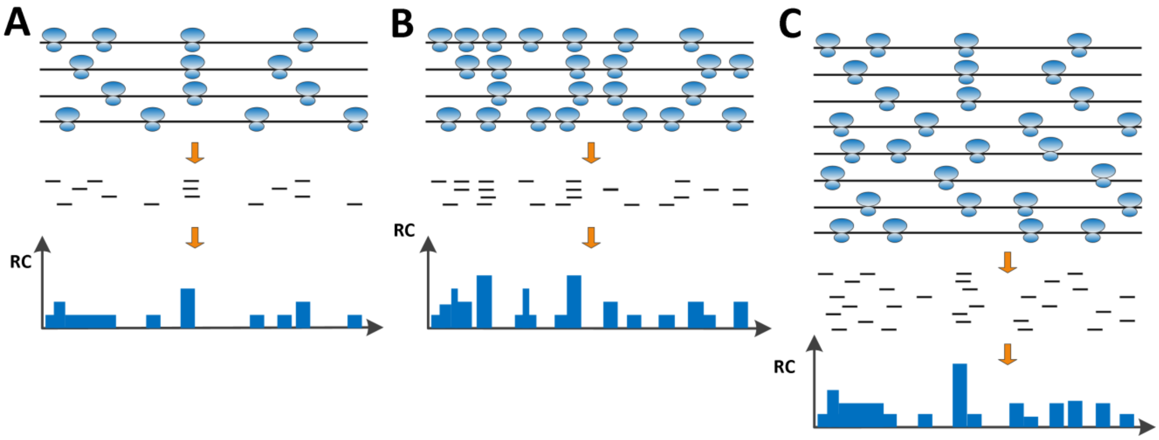

Figure 01. Demonstrating the effect of mRNA levels and initiation rate on the amount of RC. (**A**) Example of four copies of an mRNA (black lines) and a possible distribution of ribosomes on it (blue shapes). Codons that are translated more slowly on an ORF have a higher probability to be covered by ribosomes, therefore creating more ribosome protected footprints. This phenomenon is expressed in the RC profile, where slower codons have an increased number of read counts. (**B**) Higher initiation rate increases the number of ribosomes on the mRNA copies of a gene, resulting in a ribosomal profile with higher RC values. (**C**) Higher number of mRNA copies increases the number of ribosomes protected footprints, resulting in ribosomal profiles with higher RC values.

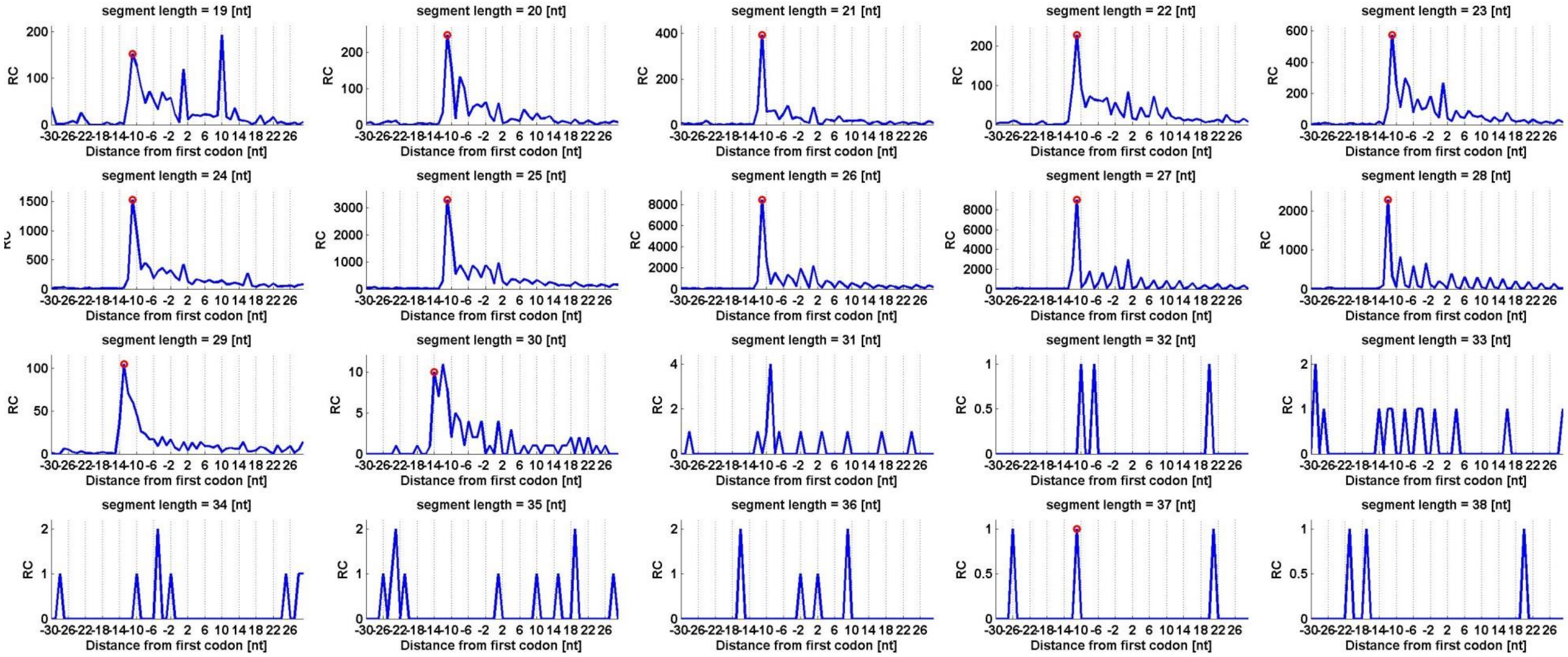

Figure 02. *S. cerevisiae* (Brar dataset) detected P values location as function of the segments length. Each subplot presents the alignment result of fragments to annotated ORFs, as function of the fragments' length. The red circles annotate the first upstream peak, associated with the offset of the fragments' 5' from the P site. Fragment groups without a definite valid peak (missing red circle) in the range of 10-25 nt upstream of the initiation site were ignored.

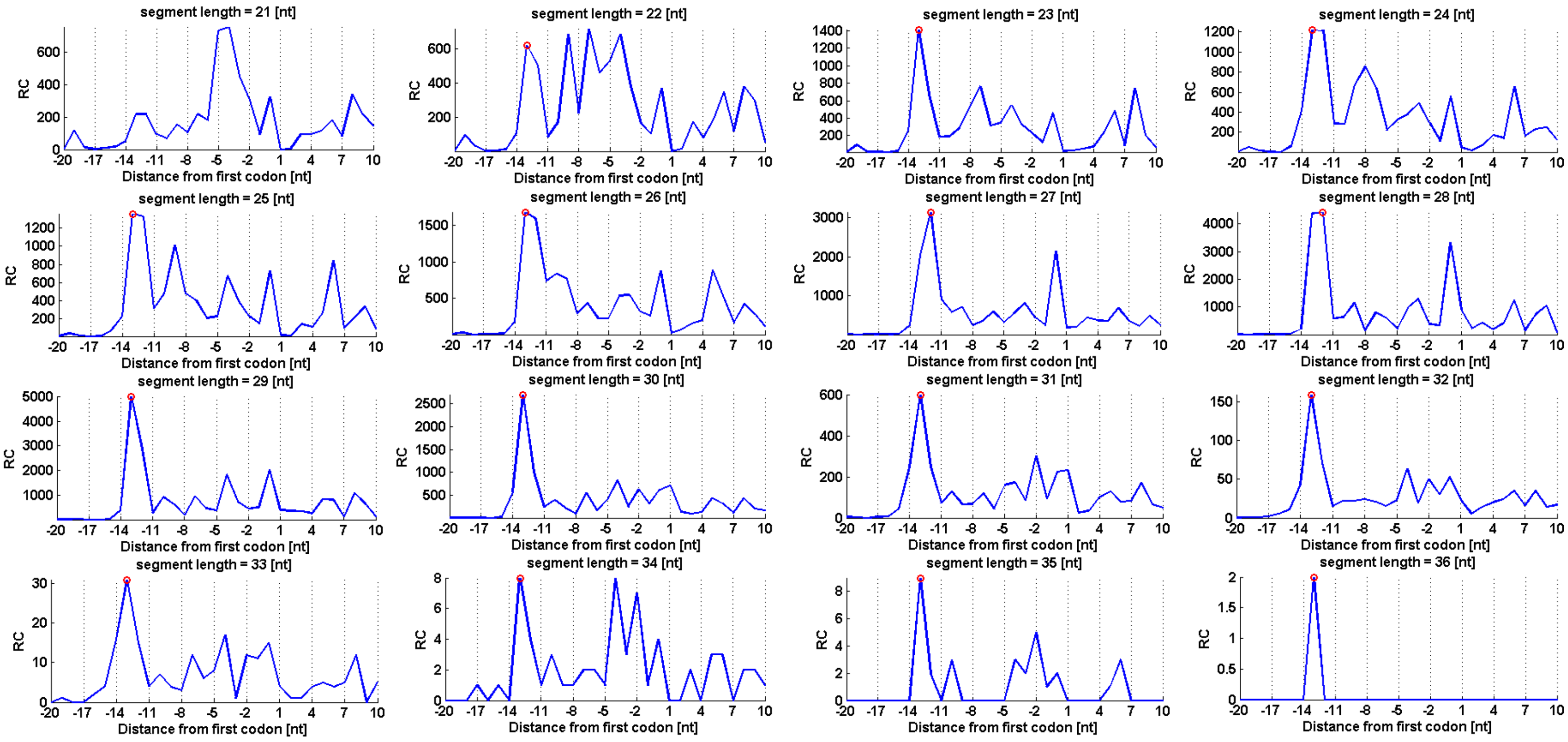

Figure 03. *S. cerevisiae* (Ingolia dataset) detected P values location as function of the segments length. Each subplot presents the alignment result of fragments to annotated ORFs, as function of the fragments' length. The red circles annotate the first upstream peak, associated with the offset of the fragments' 5' from the P site. Fragment groups without a definite valid peak (missing red circle) in the range of 10-25 nt upstream of the initiation site were ignored.

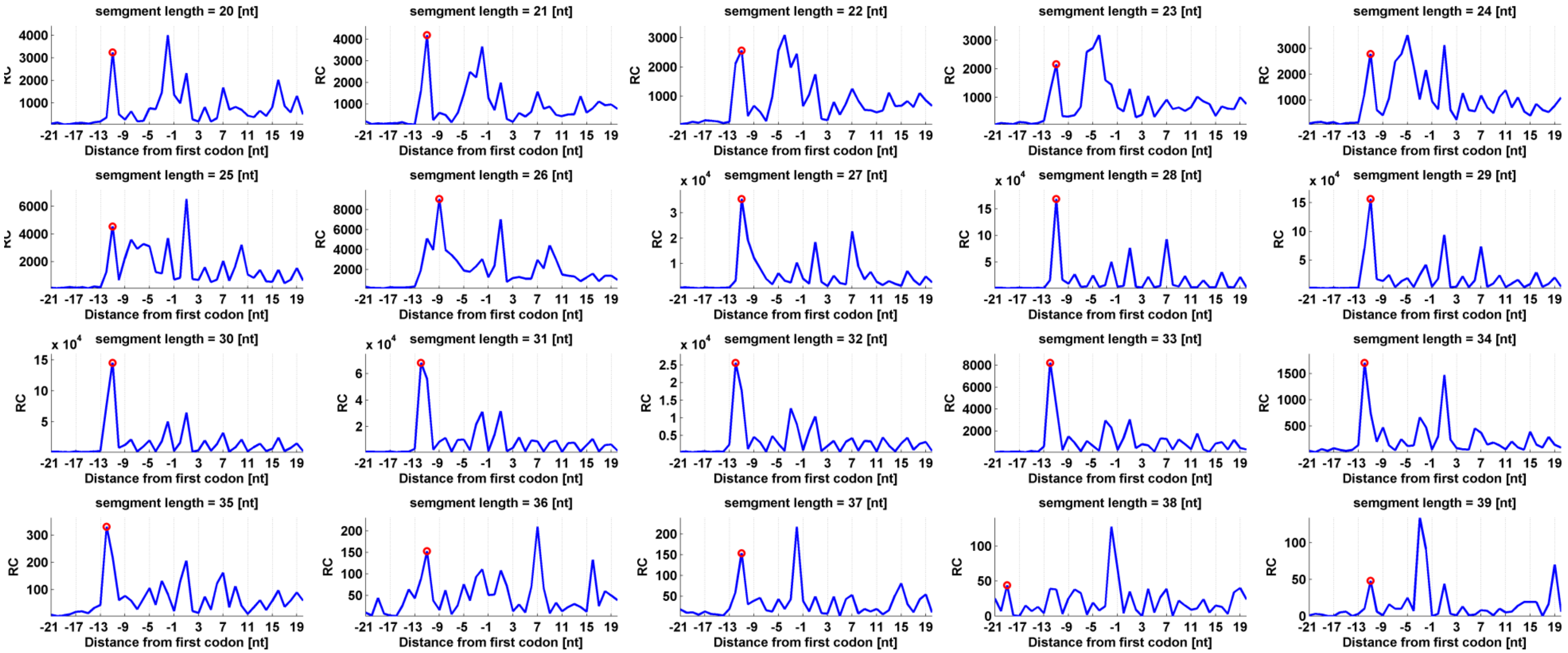

Figure 04. *C. elegans* detected P values location as function of the segments length. Each subplot presents the alignment result of fragments to annotated ORFs, as function of the fragments' length. The red circles annotate the first upstream peak, associated with the offset of the fragments' 5' from the P site. Fragment groups without a definite valid peak (missing red circle) in the range of 10-25 nt upstream of the initiation site were ignored.

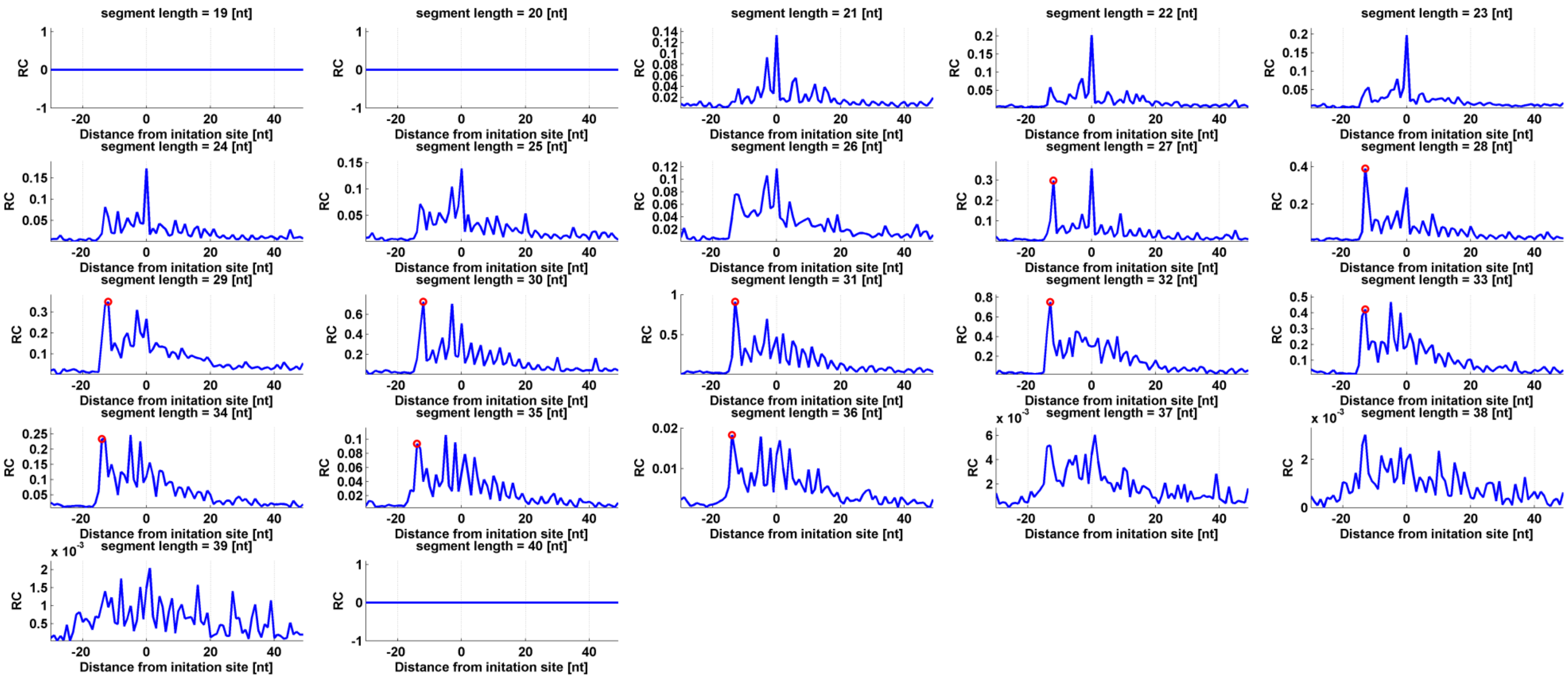

Figure 05. ***M. musculus*** **detected P values location as function of the segments length**. Each subplot presents the alignment result of fragments to annotated ORFs, as function of the fragments' length. The red circles annotate the first upstream peak, associated with the offset of the fragments' 5' from the P site. Fragment groups without a definite valid peak (missing red circle) in the range of 10-25 nt upstream of the initiation site were ignored.

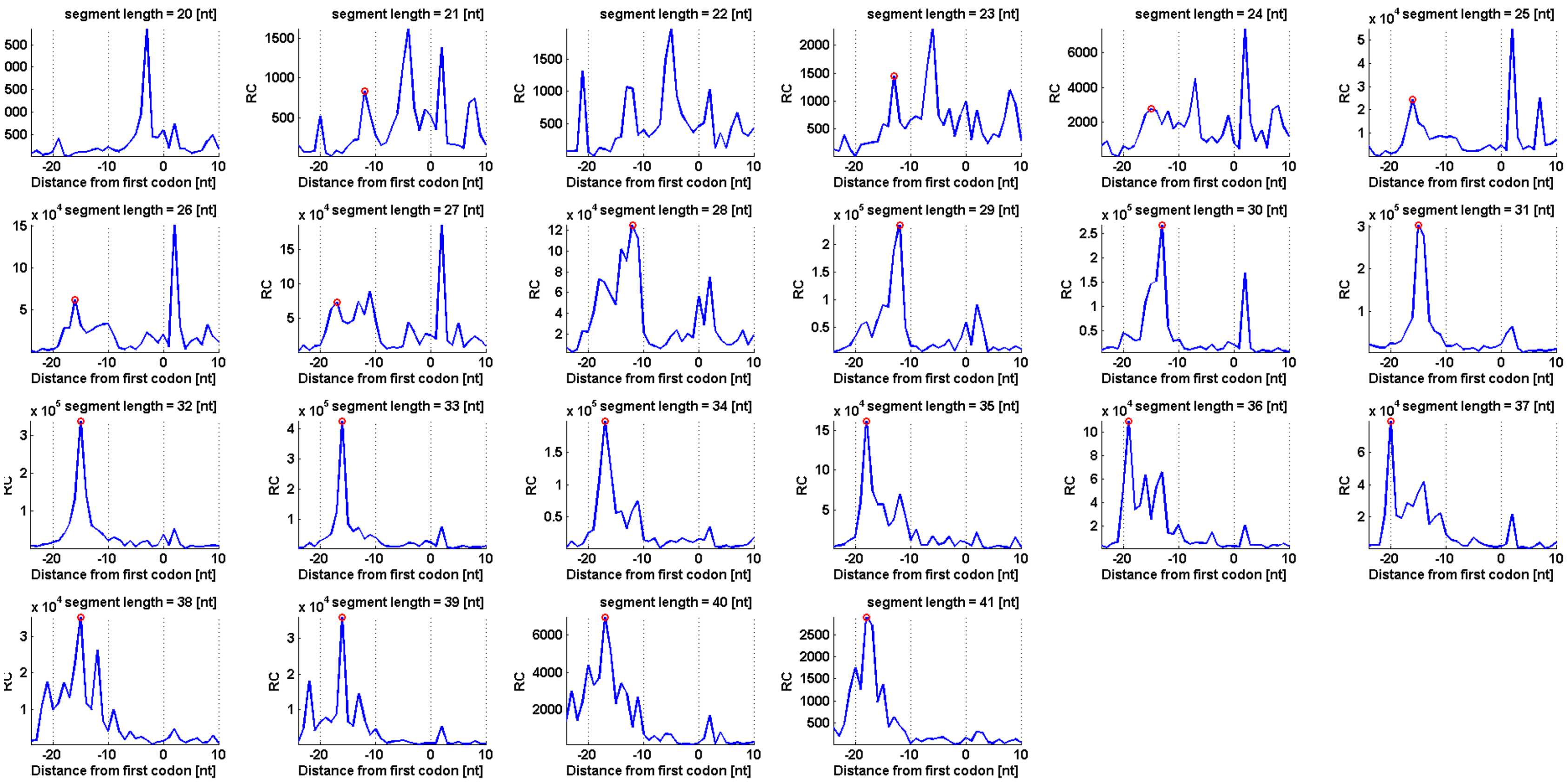

Figure 06. *E. coli* detected P values location as function of the segments length. Each subplot presents the alignment result of fragments to annotated ORFs, as function of the fragments' length. The red circles annotate the first upstream peak, associated with the offset of the fragments' 5' from the P site. Fragment groups without a definite valid peak (missing red circle) in the range of 10-25 nt upstream of the initiation site were ignored.

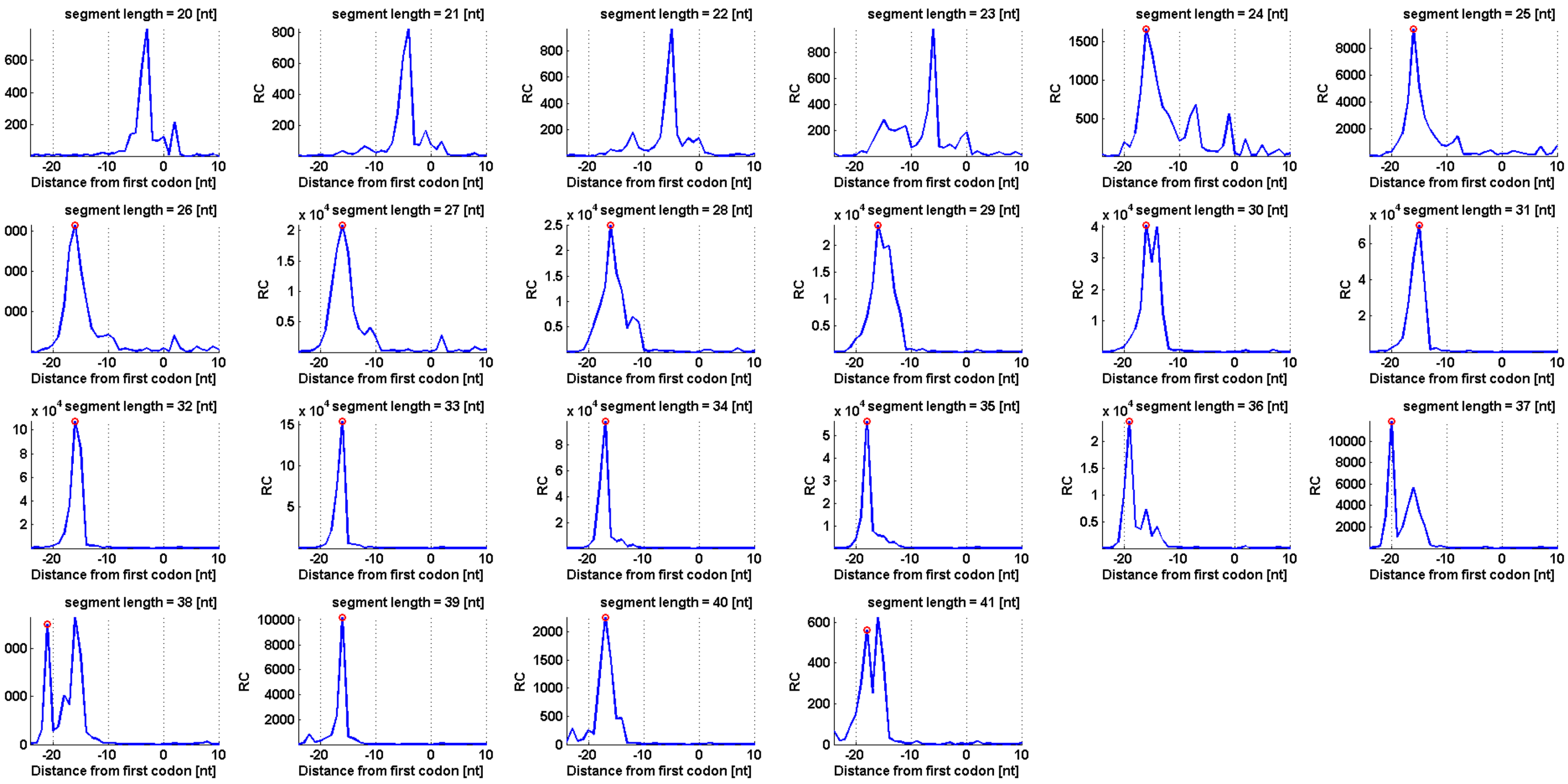

Figure 07. *B. subtilis* detected P values location as function of the segments length. Each subplot presents the alignment result of fragments to annotated ORFs, as function of the fragments' length. The red circles annotate the first upstream peak, associated with the offset of the fragments' 5' from the P site. Fragment groups without a definite valid peak (missing red circle) in the range of 10-25 nt upstream of the initiation site were ignored.

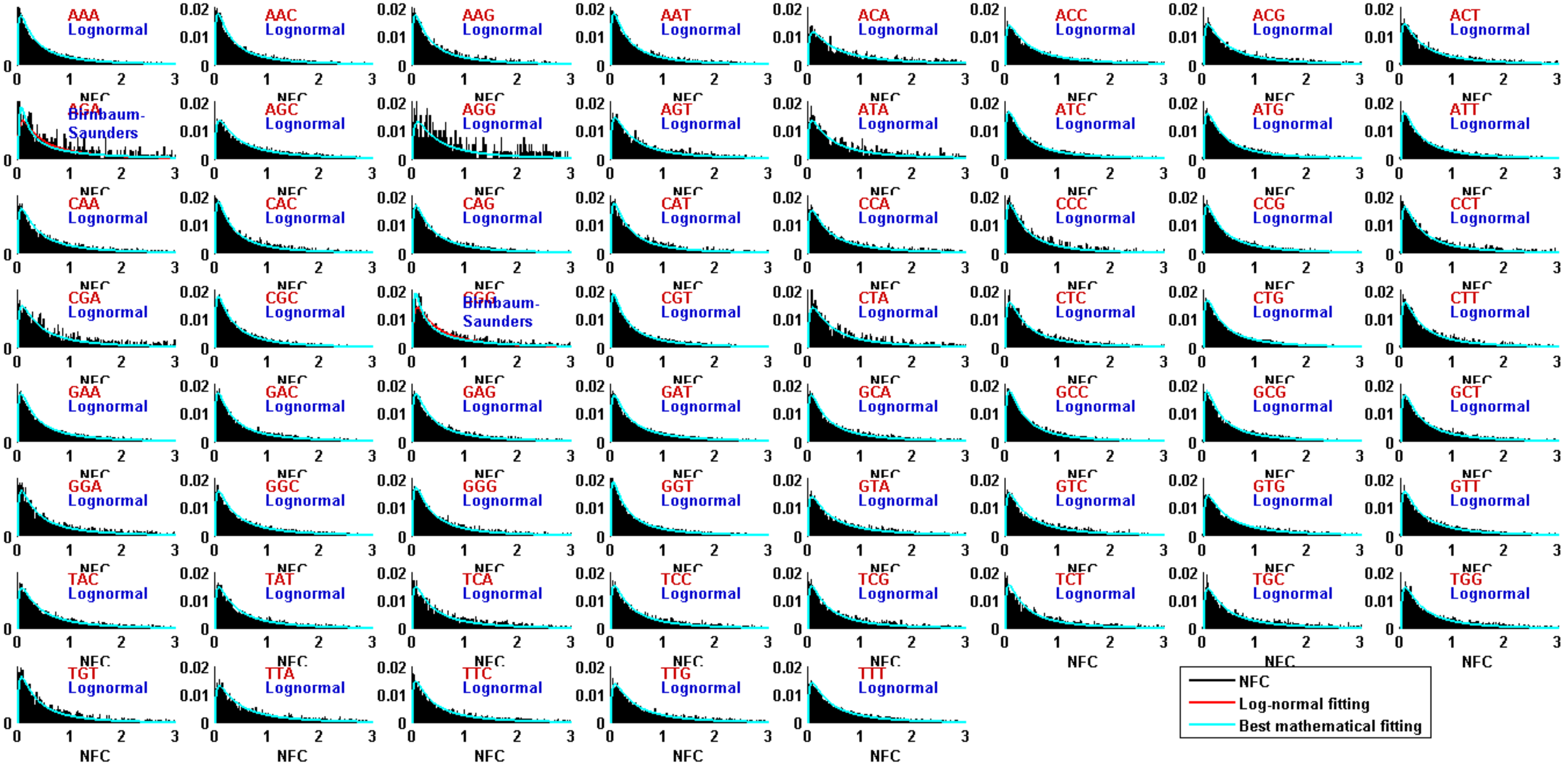

Figure 08. ***E. coli*** **codons NFC distributions.** Each subplot presents the NFC distribution of a codon (red inline text). Black histogram: NFC values; cyan: best mathematical fitting (blue inline text).

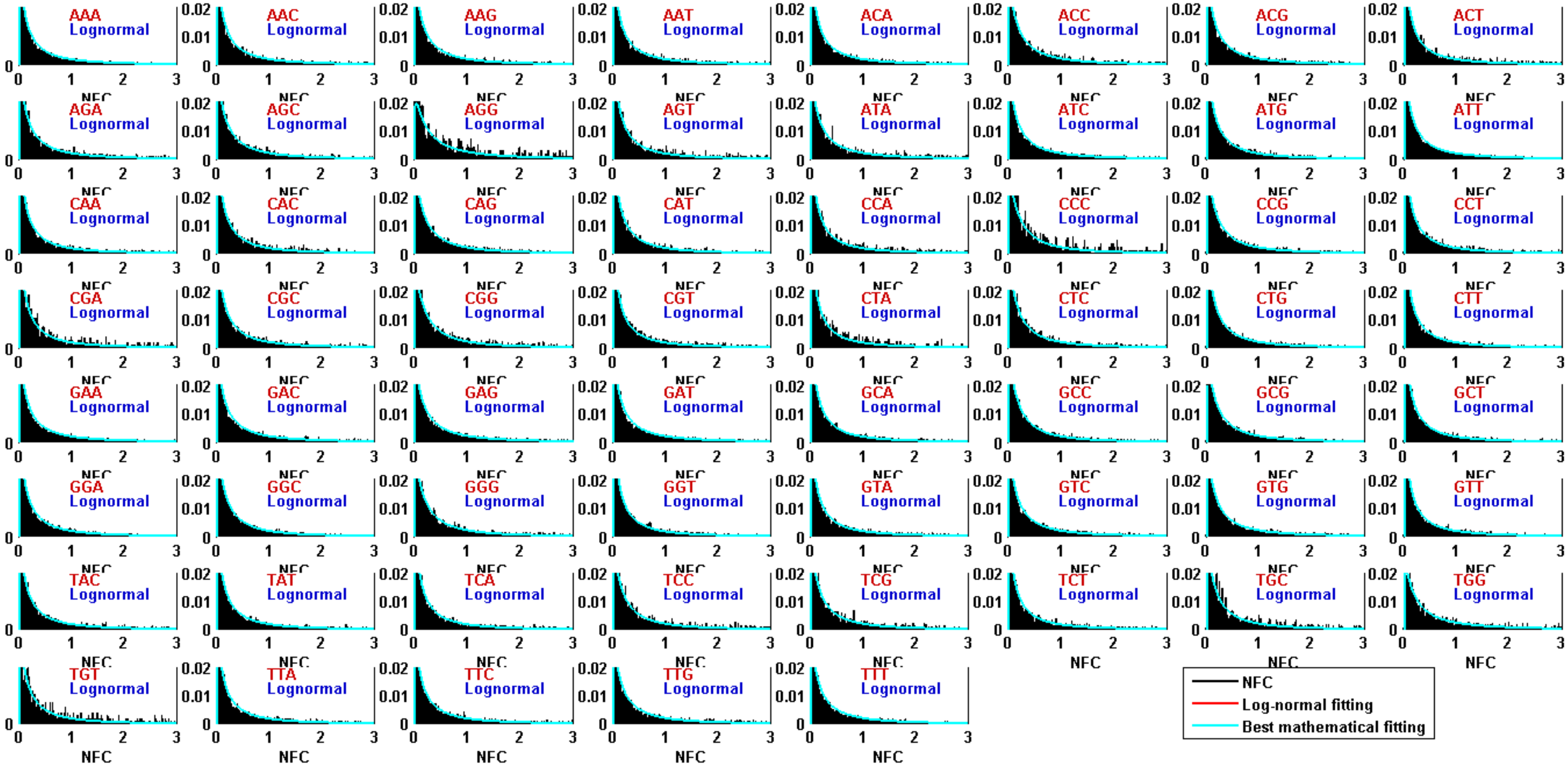

Figure 09. *B. subtilis* codons NFC distributions. Each subplot presents the NFC distribution of a codon (red inline text). Black histogram: NFC values; cyan: best mathematical fitting (blue inline text).

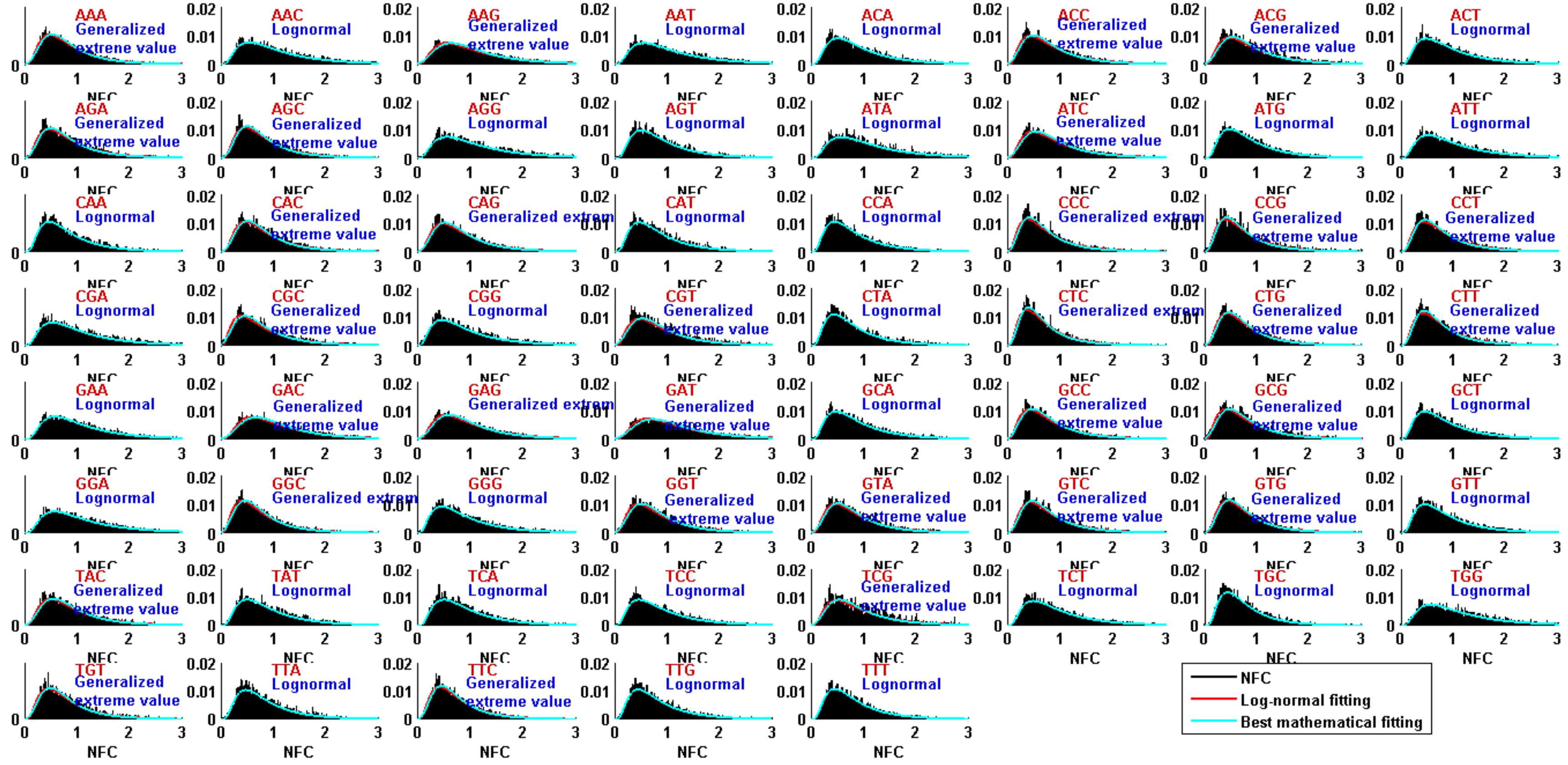

Figure 10. ***M. musculus*** **codons NFC distributions.** Each subplot presents the NFC distribution of a codon (red inline text). Black histogram: NFC values; cyan: best mathematical fitting (blue inline text).

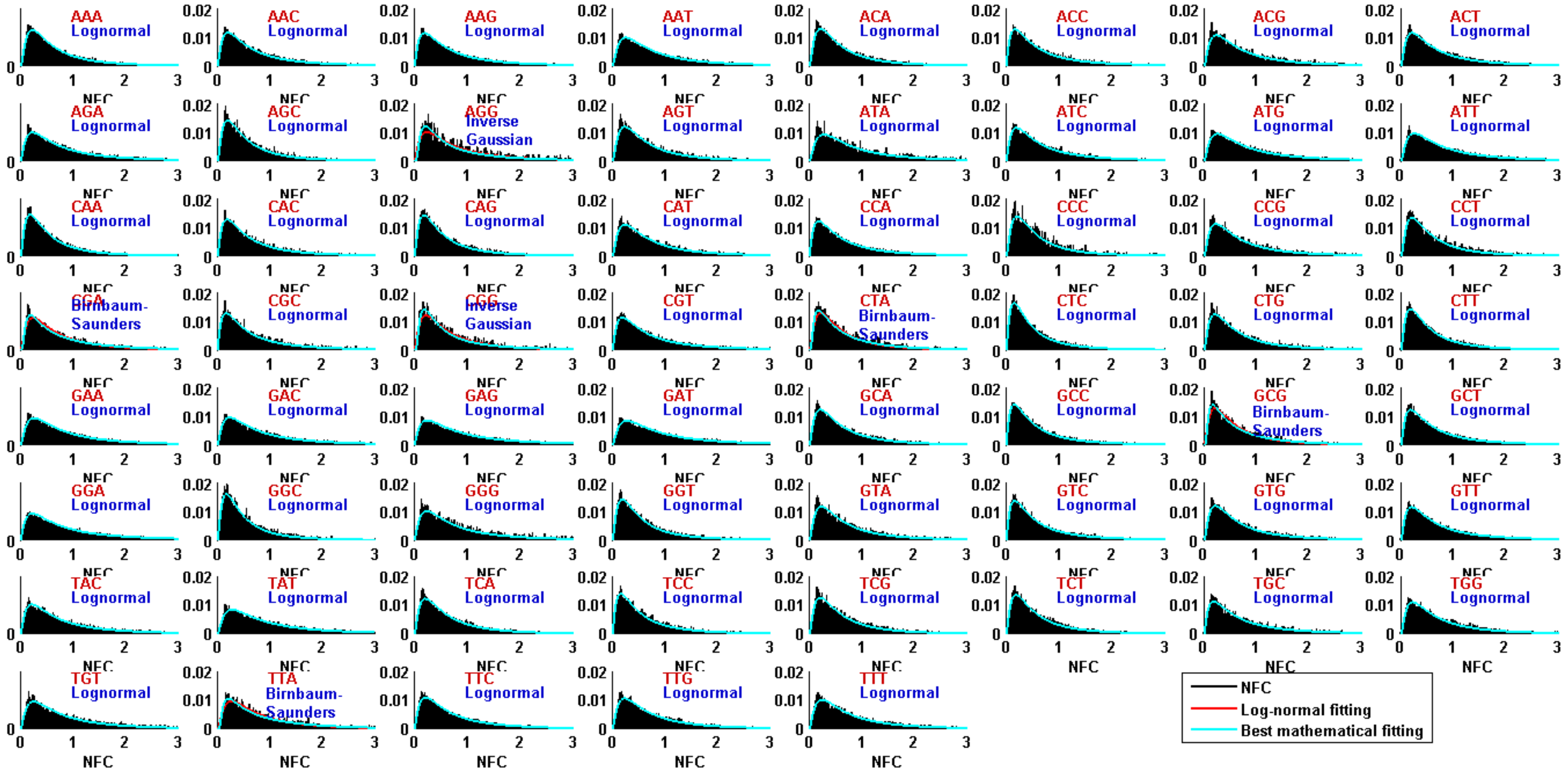

Figure 11. *C. elegans* codons NFC distributions. Each subplot presents the NFC distribution of a codon (red inline text). Black histogram: NFC values; cyan: best mathematical fitting (blue inline text).

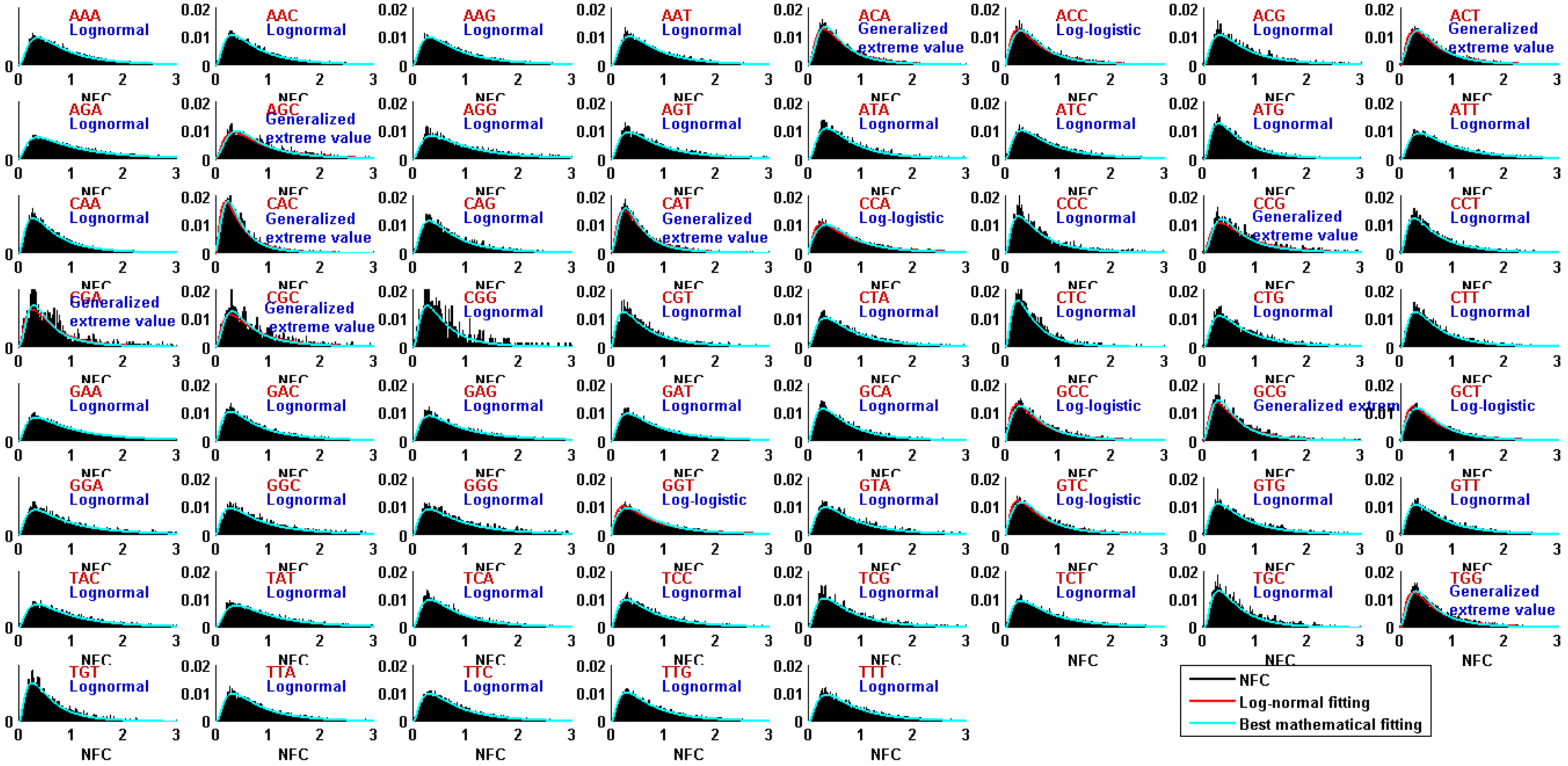

Figure 12. ***S. cerevisiae*** **codons NFC distributions.** Each subplot presents the NFC distribution of a codon (red inline text). Black histogram: NFC values; cyan: best mathematical fitting (blue inline text).

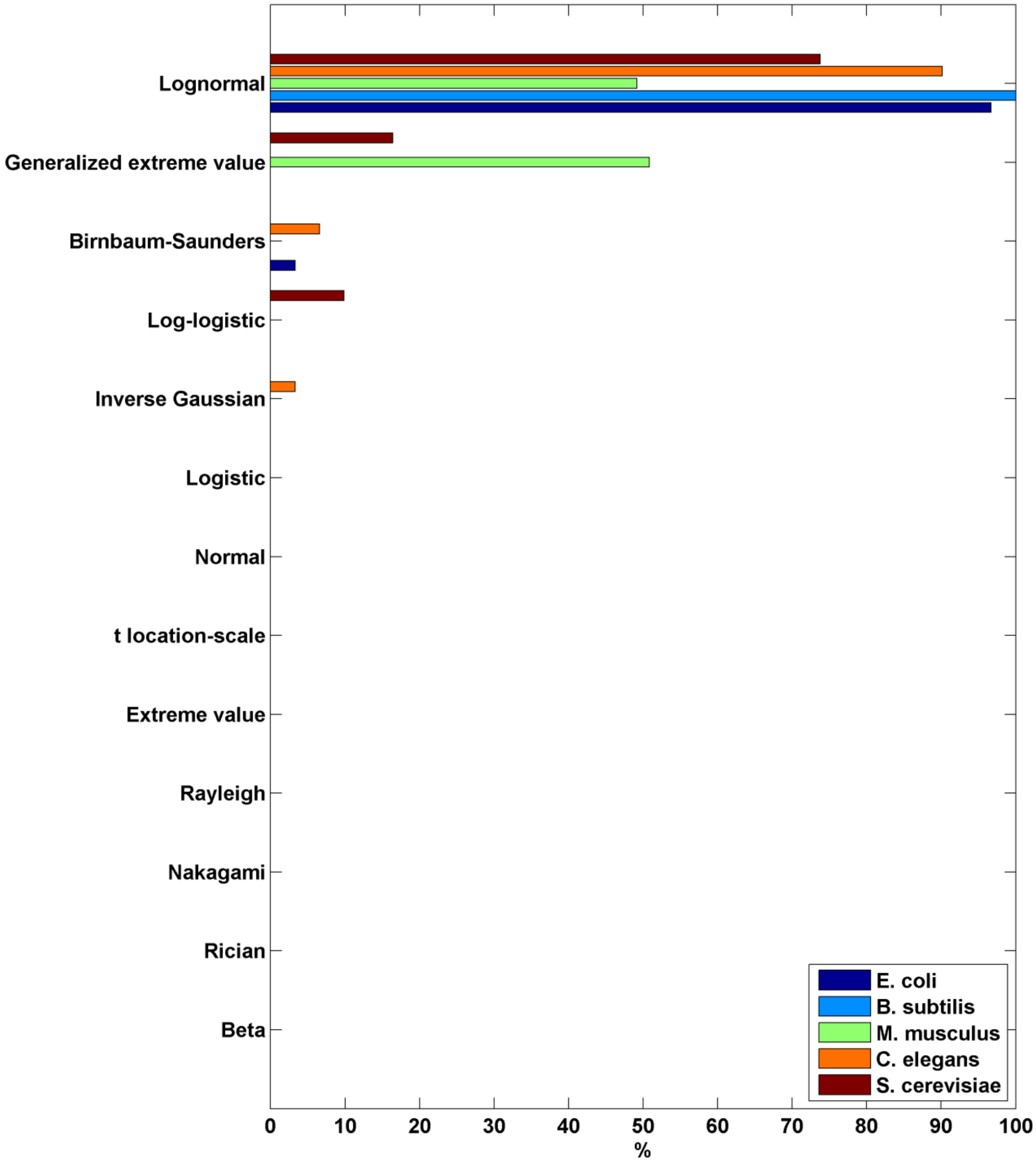

Figure 13. Distribution of various mathematical fitting functions (%) of the codons NFC distributions per organism (best fit under the log-likelihood criterion).

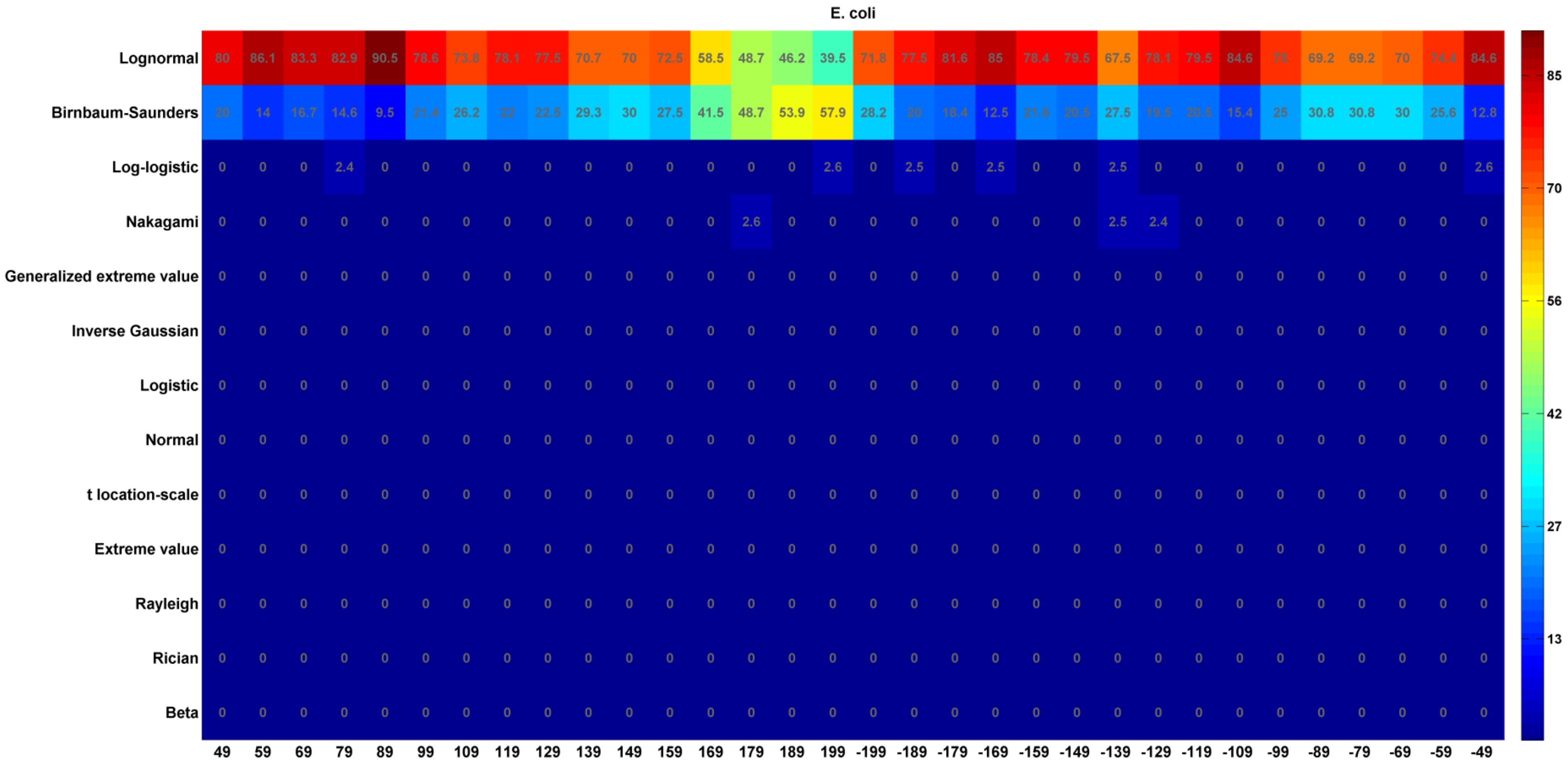

Figure 14. *E. coli* mathematical fitting of codons NFC distributions calculated on different regions of the ORF. Fittings were calculated using a sliding window of 50 codons length. The x-axis represents the location of the 3' end of the window in codon units relative to the 5' of the ORF, while negative coordinates represent the location of the 5' end of the window relative to the 3' end of the ORF. Each cell contains the percentage of codons that their NFC distributions were best fitted by the distribution types presented in the y-axis.

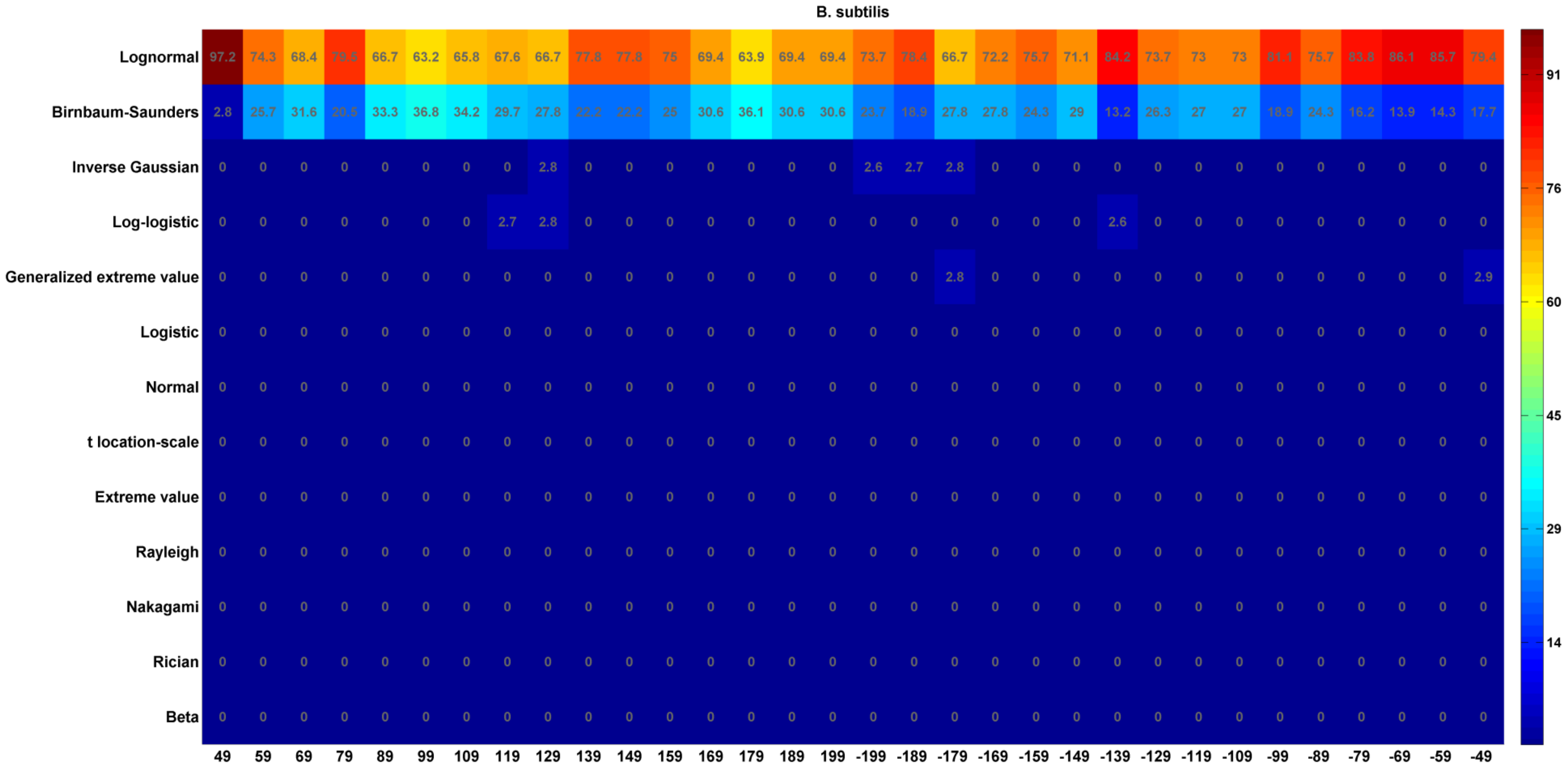

Figure 15. ***B. subtilis*** **mathematical fitting of codons NFC distributions calculated on different regions of the ORF.** Fittings were calculated using a sliding window of 50 codons length. The x-axis represents the location of the 3' end of the window in codon units relative to the 5' of the ORF, while negative coordinates represent the location of the 5' end of the window relative to the 3' end of the ORF. Each cell contains the percentage of codons that their NFC distributions were best fitted by the distribution types presented in the y-axis.

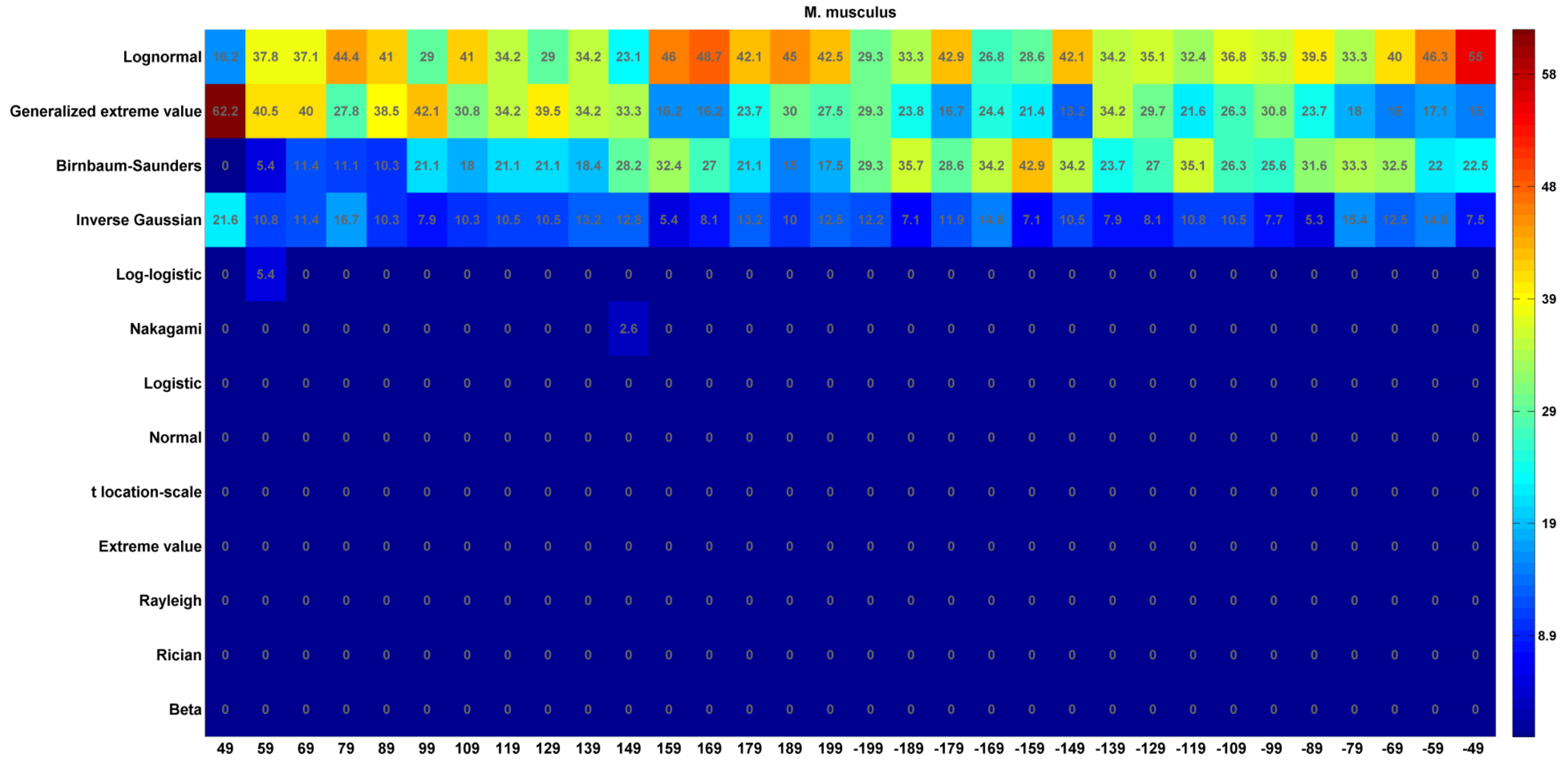

Figure 16. *M. musculus* mathematical fitting of codons NFC distributions calculated on different regions of the ORF. Fittings were calculated using a sliding window of 50 codons length. The x-axis represents the location of the 3' end of the window in codon units relative to the 5' of the ORF, while negative coordinates represent the location of the 5' end of the window relative to the 3' end of the ORF. Each cell contains the percentage of codons that their NFC distributions were best fitted by the distribution types presented in the y-axis.

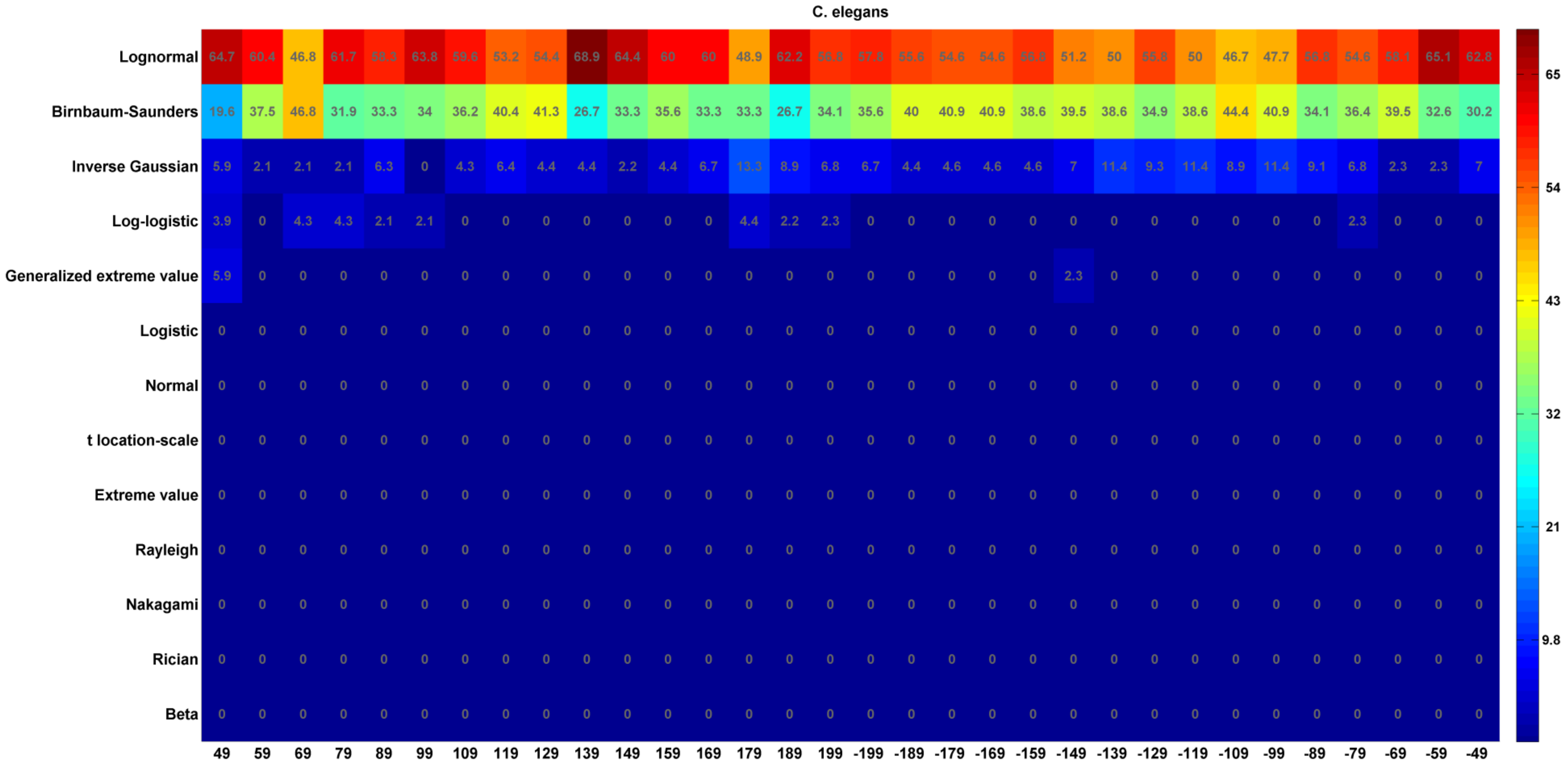

Figure 17. *C. elegans* mathematical fitting of codons NFC distributions calculated on different regions of the ORF. Fittings were calculated using a sliding window of 50 codons length. The x-axis represents the location of the 3' end of the window in codon units relative to the 5' of the ORF, while negative coordinates represent the location of the 5' end of the window relative to the 3' end of the ORF. Each cell contains the percentage of codons that their NFC distributions were best fitted by the distribution types presented in the y-axis.

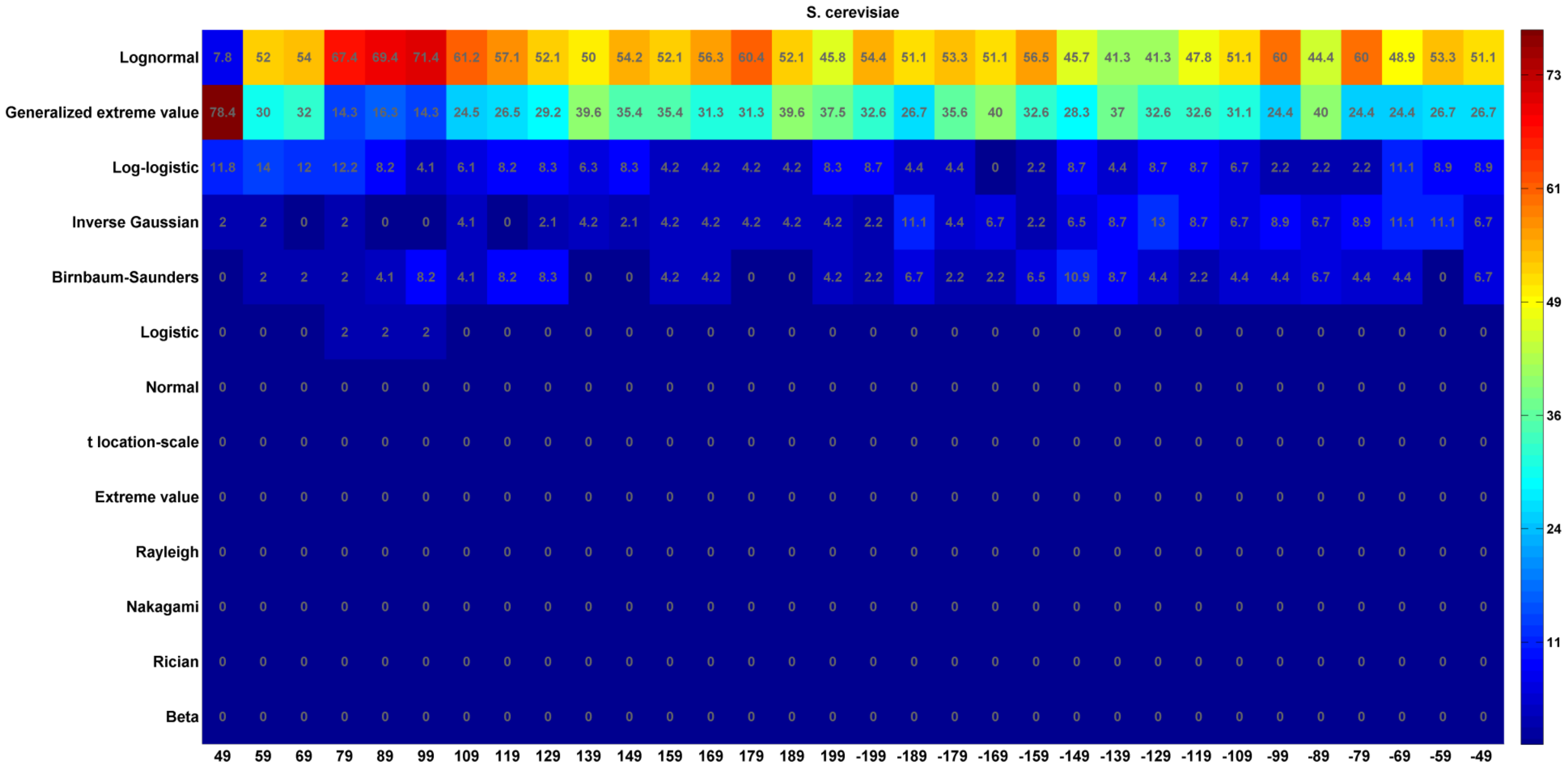

Figure 18. *S. cerevisiae* mathematical fitting of codons NFC distributions calculated on different regions of the ORF. Fittings were calculated using a sliding window of 50 codons length. The x-axis represents the location of the 3' end of the window in codon units relative to the 5' of the ORF, while negative coordinates represent the location of the 5' end of the window relative to the 3' end of the ORF. Each cell contains the percentage of codons that their NFC distributions were best fitted by the distribution types presented in the y-axis.

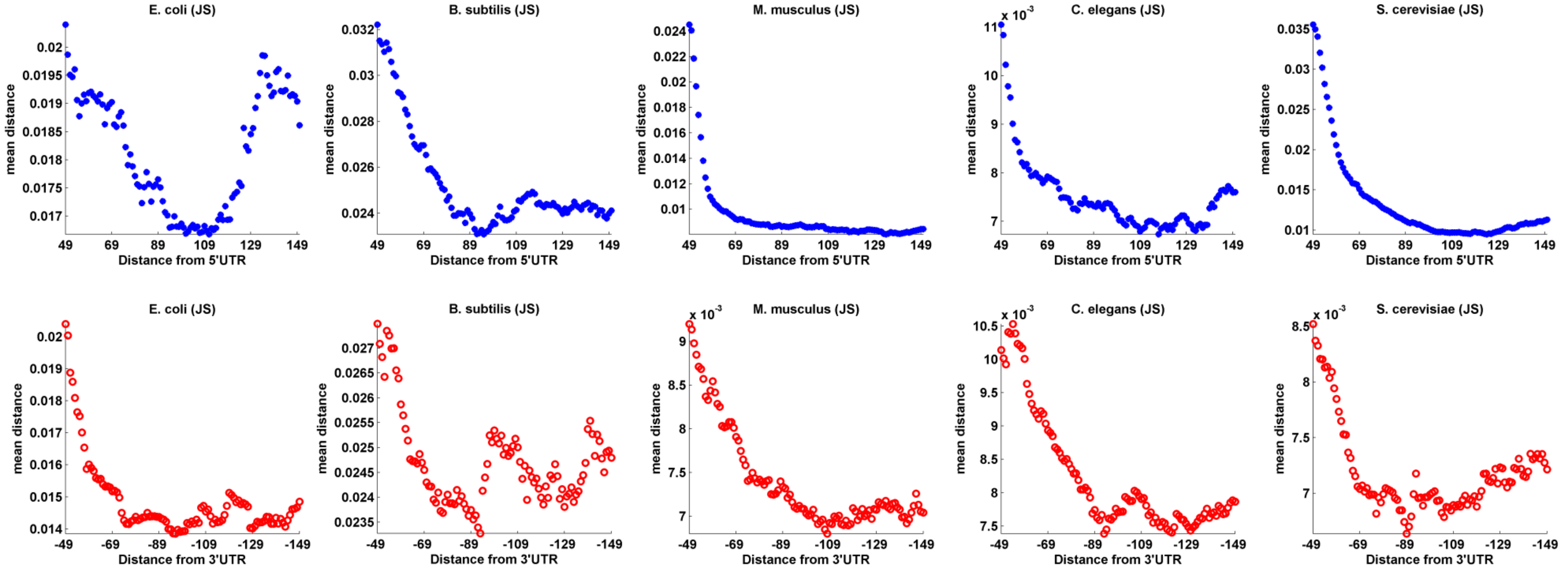

Figure 19. Mean distance vector calculated using the JS distance metric. The mean distance vector represents the average distance between a NFC distribution calculated in a specific window to all the other windows (across all codons). The upper blue graphs represent the mean distance vector calculated for the first 101 windows (window length of 50 codons), while the bottom red graphs represent the mean distance vector calculated for the last 101 windows. The x axis of the top/bottom graphs represents the distance between the 3'/5' end of the window in codon units from the 5'/3' end of the ORFs respectively (negative distance represents distance from the ORF's 3').

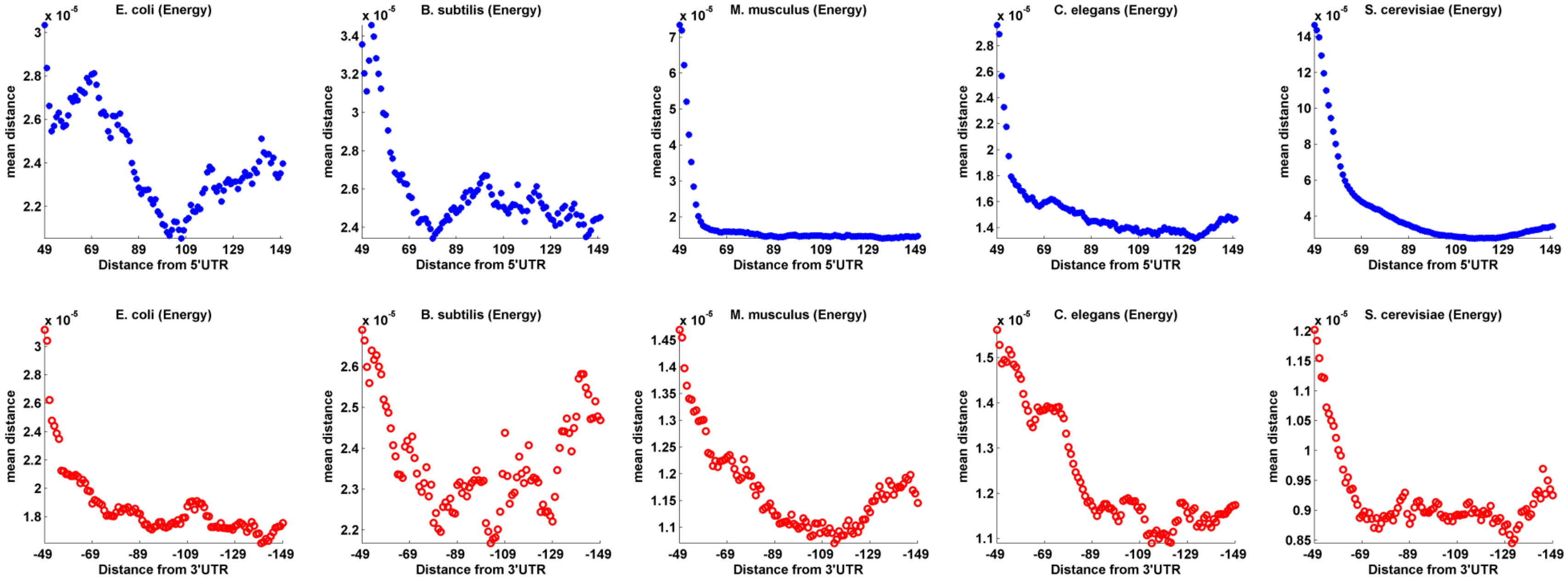

Figure 20. Mean distance vector calculated using the Energy distance metric. The mean distance vector represents the average distance between a NFC distribution calculated in a specific window to all the other windows (across all codons). The upper blue graphs represent the mean distance vector calculated for the first 101 windows (window length of 50 codons), while the bottom red graphs represent the mean distance vector calculated for the last 101 windows. The x axis of the top/bottom graphs represents the distance between the 3'/5' end of the window in codon units from the 5'/3' end of the ORFs respectively (negative distance represents distance from the ORF's 3').

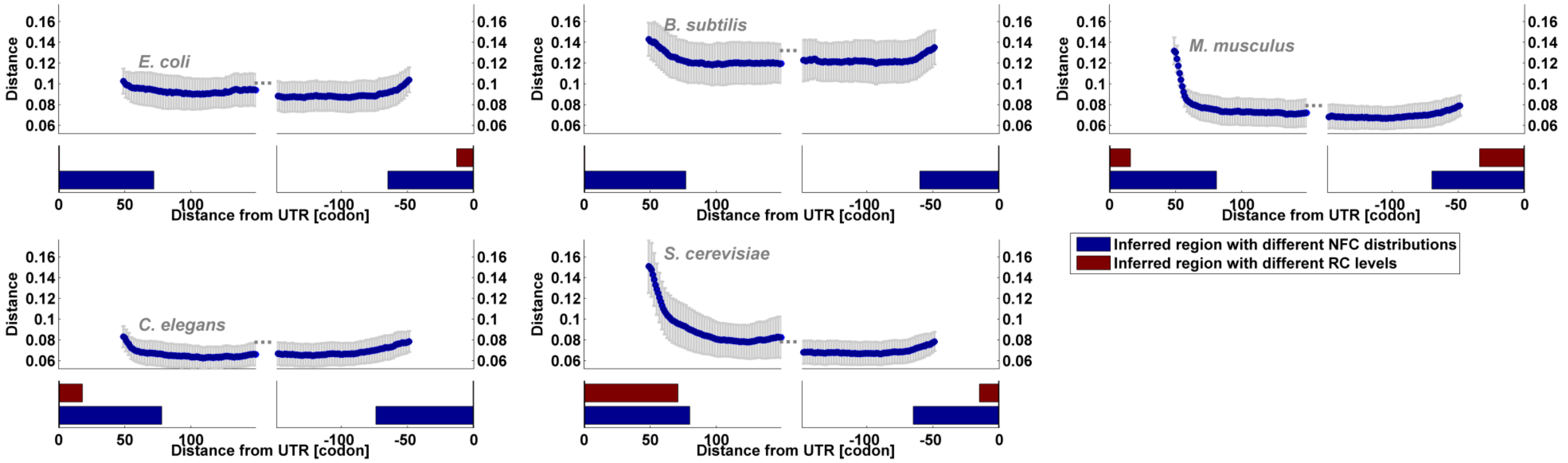

Figure 21. Comparison of the mean distance among different organisms. Each subplot describes the mean distance vector calculated on the first and last 100 windows using the Hellinger metric (dotted graphs). The navy bars beneath mark the regions relatively to the 5'/3' end with significantly different NFC distributions in comparison to subsequent regions on the ORFs. A similar test was directly applied on the averaged RC profiles (instead on the mean distance vector; see Figure S24), shown in burgundy bars (absent bars indicate of no such region). For a comparison between organisms, all plots are identically scaled.

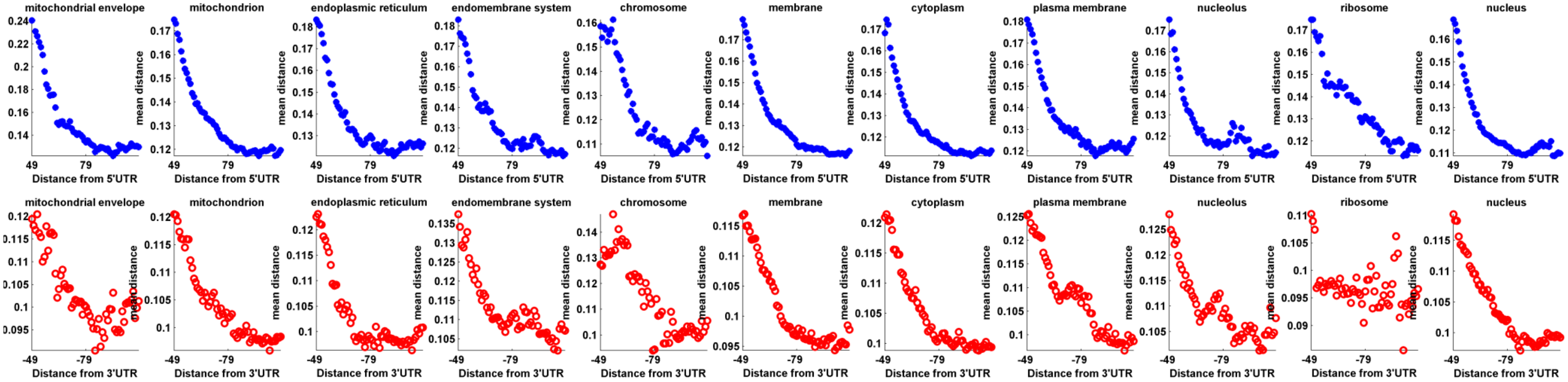

Figure 22. Mean distance vector calculated using the Hellinger distance metric for different *S. cerevisiae* cellular components. The mean distance vector represents the average distance between a NFC distribution calculated in a specific window to all the other windows (across all codons). The upper blue graphs represent the mean distance vector aligned to ORFs 5' end while the bottom red graphs represent the mean distance vector to windows aligned to ORFs 3' end. The x axis of the top/bottom graphs represents the distance between the 3'/5' end of the window in codon units from the 5'/3' end of the ORFs respectively (negative distance represents distance from the ORF's 3').

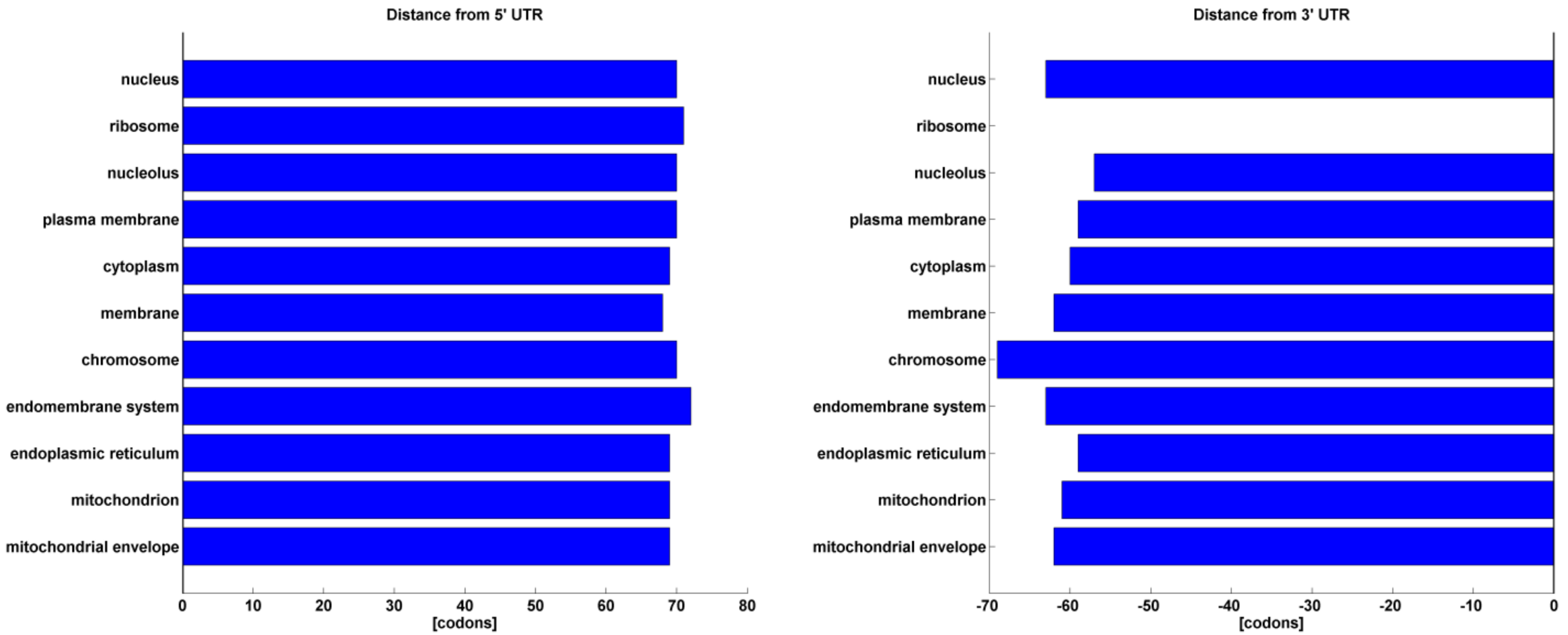

Figure 23. Regions characterized by different codons NFC distributions, estimated using the Hellinger distance metric for different *S. cerevisiae* GO cellular components. The regions were calculated (relatively to the 5'/3' end of the ORFs), based on the mean distance vector presented in Figure S17.

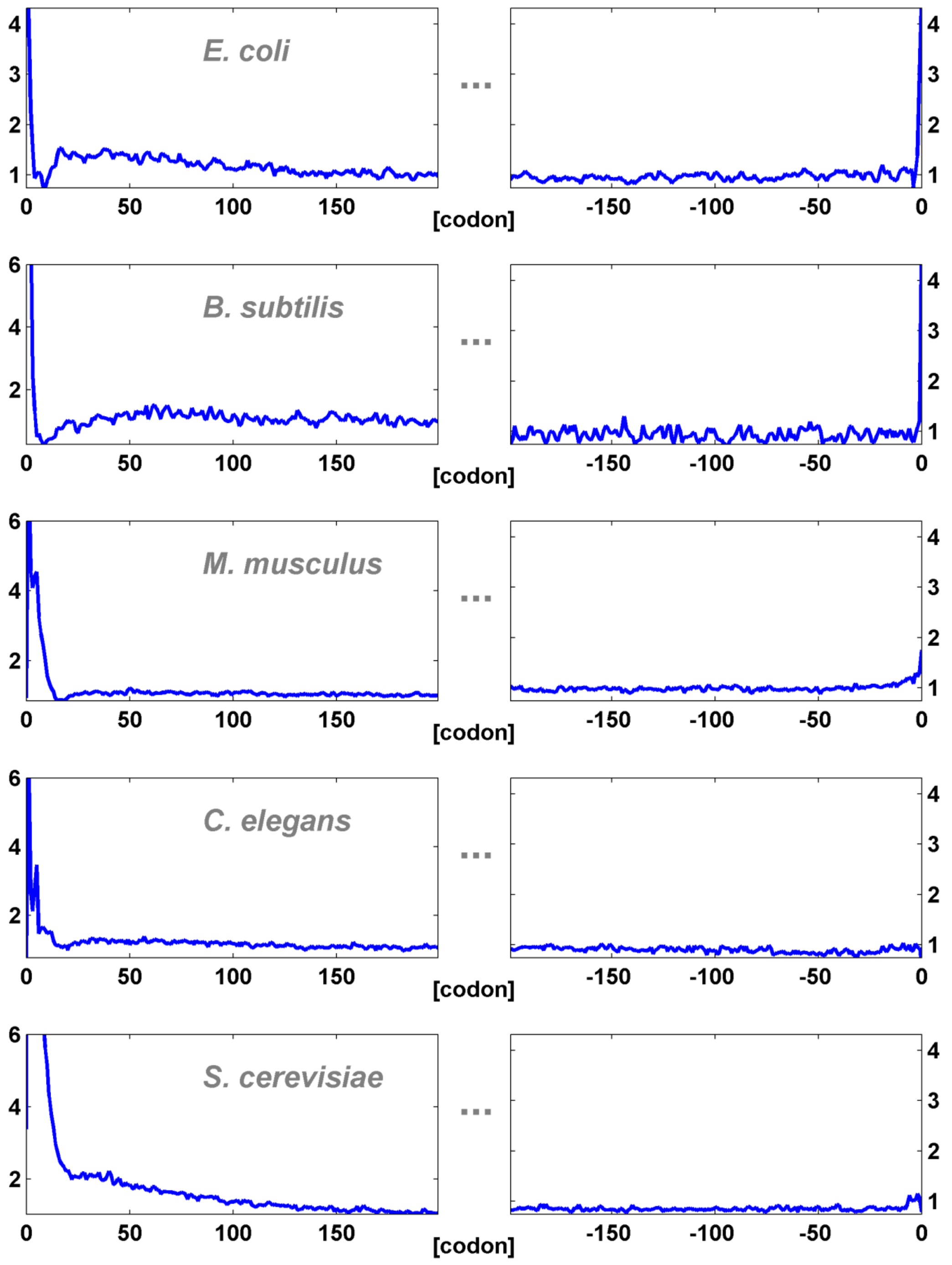

Figure 24. Mean RC profiles of genes selected for analysis in the studied organisms. Prior to averaging, RC of each gene were normalized by its mean RC, excluding the first and last 60 codons [2]. Only genes of at least 400 codons length were included in this presentation.

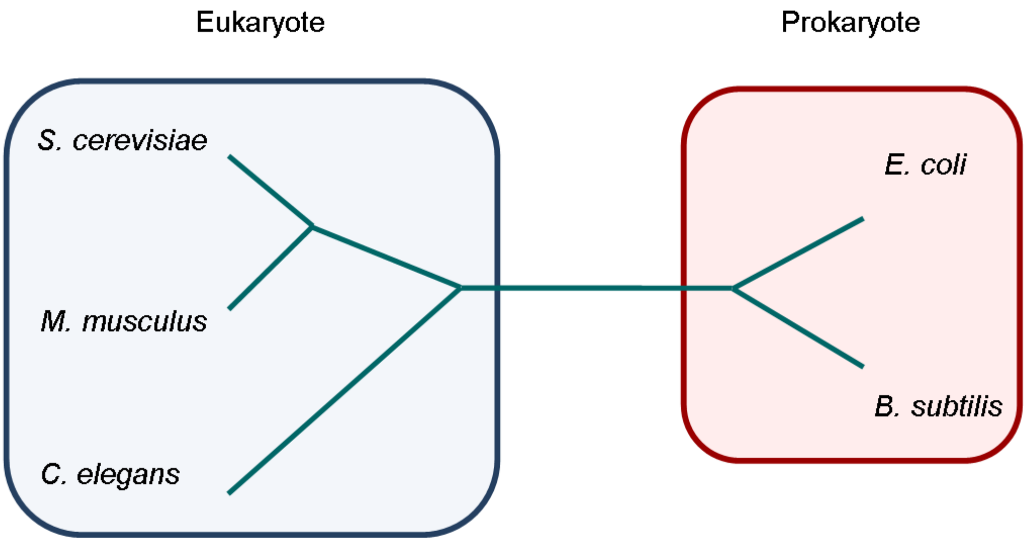

Figure 25. Clustering organisms according to the distance between NFC distributions across organisms. NFC distributions were averaged over all codon types and clustered using the neighbor joining algorithm. The same structure was obtained when using the different distance metrics.

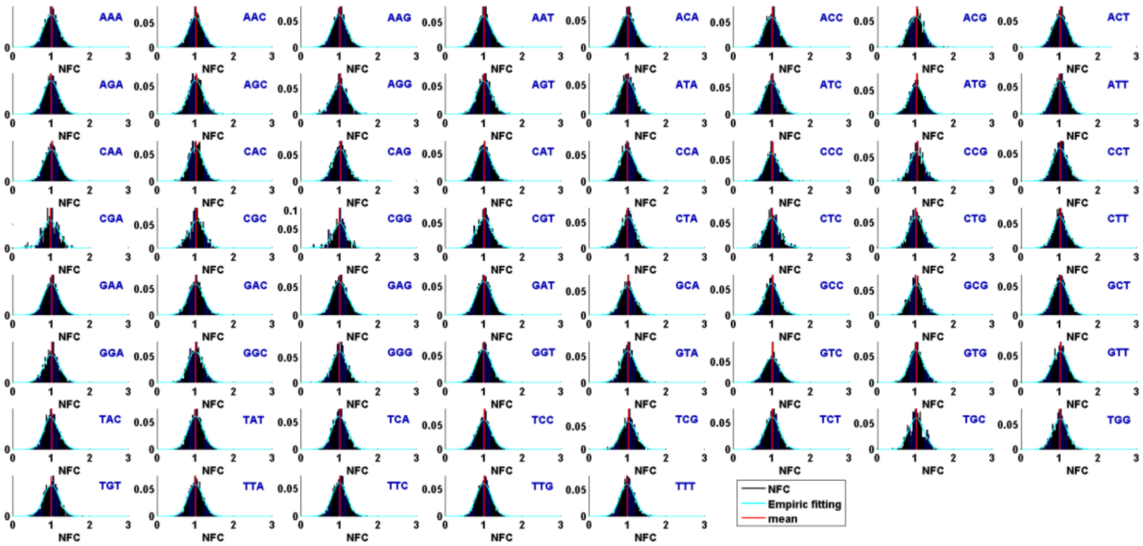

Figure 26. Demonstrating that when no ribosome interactions are present, the NFC distributions are normally distributed. *S. cerevisiae* ribosome profiles were simulated using the TASEP process. All codon decoding times were set to be exponentially distributed with a rate λ = 1 and initiation rate was set to 0.3. Ribosome profiles of each gene were simulated using 500 mRNA copies. The black histograms depict NFC values of each codon type, collected from all simulated genes; the cyan curve represents the empirical fitting of the NFC distributions, while the red line represents the mean NFC value for each codon type. NFC values are normally distributed (under KS-test: mean p value = 0.27).

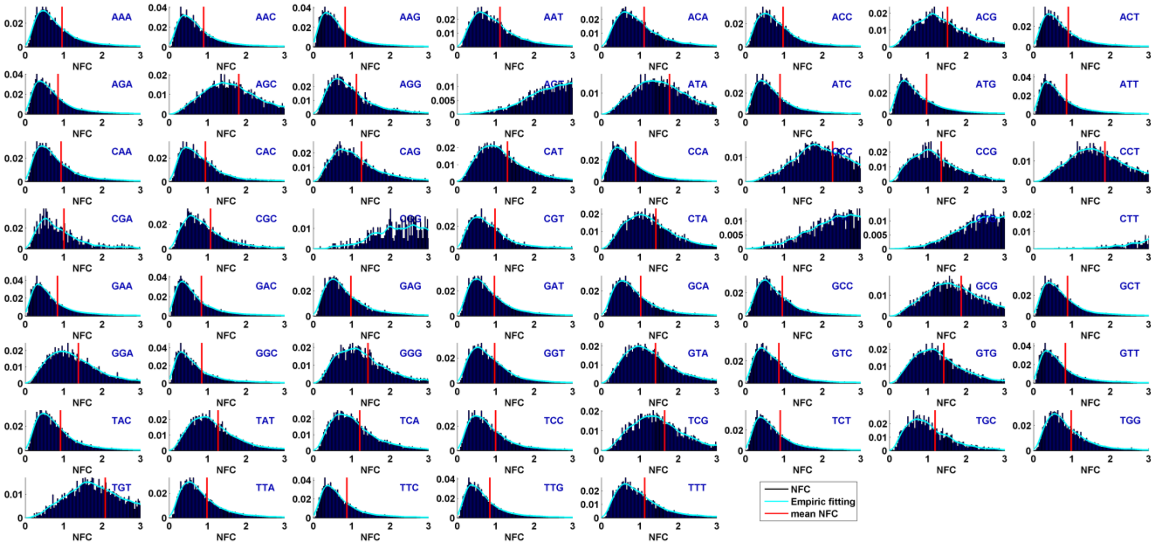

Figure 27. *S. cerevisiae* ribosomal profiles were simulated using the TASEP process. The black histograms depict NFC values of each codon type, collected from all simulated genes; the cyan curve represents the empirical fitting of the NFC distributions, while the red line represents the mean NFC value for each codon type.

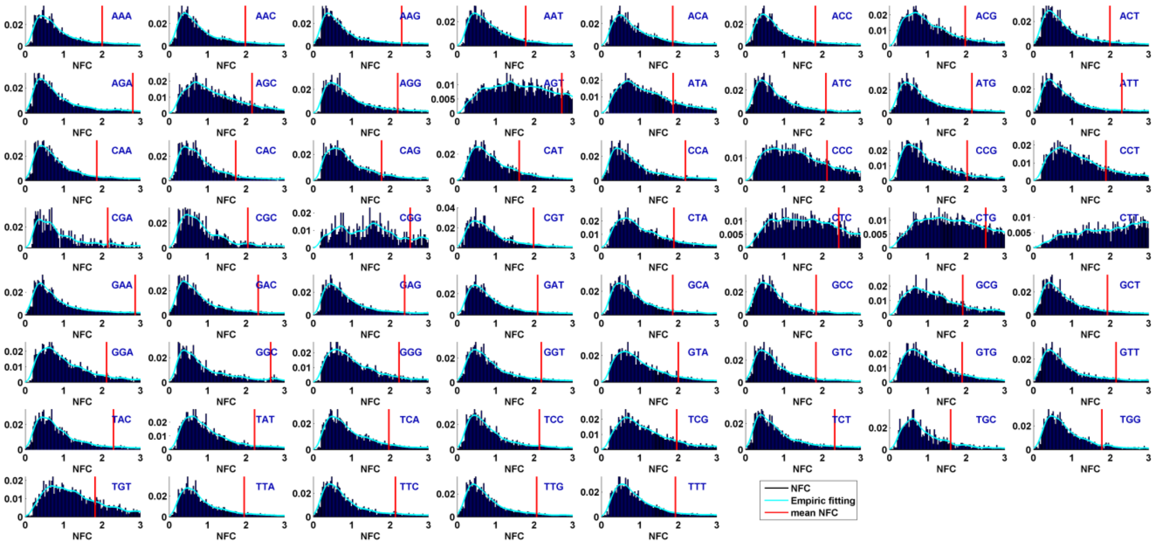

Figure 28. Simulating the influence of translational pauses on *S. cerevisiae* ribosomal profiles. Location of translational pauses were set according to the location of the translational pauses in the real *S. cerevisiae* ribosomal profiles. The black histograms depict NFC values of each codon type, collected from all simulated genes; the cyan curve represents the empirical fitting of the NFC distributions, while the red line represents the mean NFC value for each codon type.

Supplementary Tables

Table 01. Alignment statistics of the analyzed organisms. Each row depicts the statistics of each processed replica of an organism. The second column depicts the number of obtained fragments from each replica, after filtering fragments of a quality lower than 50. The third column depicts the number of fragments after linker removal (see specific linker details in Supplementary Methods 'Reconstructing ORFs ribosomal profiles of the analyzed organisms') that had at least 19 nt. The fourth column depicts the number of remaining fragments after filtering fragments that aligned to rRNA sequences. The fifth column represents the number of fragments that aligned to annotated ORFs and spliced junctions. The sixth column describes the percentage of fragments that their P site could not be determined (details in Supplementary Methods 'Reconstructing ORFs ribosomal profiles of the analyzed organisms'). Columns seven – ten describe the percentage of remaining fragments that were mapped to one/two/three/more than three locations.

Source ID	# fragments after quality check filtering	# fragments after linker removal	#fragments after linker removal, no rRNA	#fragments aligning to ORFs and spliced junctions	%ignored fragments (P site not determined)	%fragments aligning to one location	%fragments aligning to two locations	%fragments aligning to three locations	%fragments aligning to more than three locations
E. coli GSM872393	364,847,208	354,076,633	183,806,983	173,814,062	0.27	90.92	9.06	0.02	0.00
E. coli GSM872394	291,698,307	291,698,307	133,053,267	125,850,650	0.27	91.46	8.52	0.02	0.00
B. subtilis GSM872395	18,230,224	16,609,107	7,109,618	6,294,848	0.84	91.02	8.98	0.00	0.00
B. subtilis GSM872397	212,302,107	187,999,657	79,377,192	67,203,910	0.51	92.06	7.94	0.00	0.00
M. musculus GSM765292	41,770,345	31,597,630	22,921,785	12,628,422	20.51	79.12	13.90	1.74	5.24
C. elegans SRA055804 (SRR522883)	146,812,635	133,099,603	38,179,130	32,879,867	0	77.78	16.49	1.76	3.97
S. cerevisiae Ingolia GSM346111	12,636,882	12,165,707	2,502,888	1,654,685	8.52	72.79	24.92	1.52	0.77
S. cerevisiae	23,899,689	23,671,303	13,515,244	2,936,633	34.82	73.56	24.30	1.36	0.78

Ingolia GSM346114									
S. cerevisiae Brar GSM843780	147,785,344	147,785,050	30,110,393	10,855,927	6.81	93.46	6.13	0.28	0.13
S. cerevisiae Brar GSM843781	128,476,594	128,476,338	32,016,710	11,082,229	4.40	93.63	5.97	0.27	0.13

Table 02. Used thresholds per organism for determining if the RC profile of a gene had enough RC to further participate in the analysis. Genes with lower RC than described in the second column were excluded. The third column depicts the number of genes participating in the analysis.

Organism	Minimal median RC per ORF	Number of analyzed genes
E. coli	1	2832
B. subtilis	1	1862
M. musculus	1	2991
C. elegans	1	3959
S. cerevisiae (Brar)	1	1808
S. cerevisiae (Ingolia)	1	1189

Table 03. Determining the significance of NFC distribution uniqueness. self-distance and of the NFC distribution was compared to NFC distributions of other codon types (A) /of other codon types with nucleotide content (B) / of other codon types coding for the same amino acids (C).

Organism	JS			Hellinger			Energy		
	A	B	C	A	B	C	A	B	C
E. coli	0	0.02	0	0	0.02	0.02	0	0.03	0
B. subtilis	0	0.15	0.04	0	0.17	0.17	0	0.2	0.04
M. musculus	0	0	0	0	0	0	0	0	0
C. elegans	0	0	0	0	0	0	0	0	0
S. cerevisiae	0	0	0	0	0	0	0	0	0

Table 04. Regions (relatively to 5'/3' end of ORFs) characterized by different codons NFC distributions, estimated using different distance metrics, when considering all RC in the window, for all codon types.

	Distance from 5' UTR [codons]			Distance from 3' UTR [codons]		
	JS	Hellinger	Energy	JS	Hellinger	Energy
E. coli	62	72	85	64	65	66
B. subtilis	78	77	72	58	60	58
M. musculus	76	81	78	71	70	72
C. elegans	74	78	83	73	74	76
S. cerevisiae	77	80	79	60	65	59

Table 05. Average regions lengths (relatively to 5'/3' end of ORFs) characterized by different codons NFC distributions, estimated using different distance metrics, when considering all RC in the window, for all codon types. The regions were calculated for sliding windows of 40/50/60 codons and a depth of the first and last 160/180/200/220/240 codons relatively to the 5'/3' ends of the ORFs.

	Distance from 5' UTR [codons]			Distance from 3' UTR [codons]		
	JS	Hellinger	Energy	JS	Hellinger	Energy
E. coli	73.30+/-10.33	69.27+/-13.91	77 +/-16.93	60.40+/-10.75	63.07+/-10.88	65.73+/-8.27
B. subtilis	74.40+/-8.32	74.67+/-7.75	71.00+/-9.55	58.73+/-12.41	60.87+/-12.12	58.20+/-12.54
M. musculus	76.27+/-8.84	80.40+/-8.34	74.87+/-11.35	70.07+/-9.97	69.40+/- 9.66	70.60+/-12.29
C. elegans	74.27+/-11.90	75.27+/-10.78	79.67+/-12.02	60.33+/-9.29	73.07+/- 8.08	72.87+/-9.17

S. cerevisiae	78.47+/- 9.46	79.87 +/-9.64	79.40+/-10.11	63.07+/-10.88	64.07+/- 9.07	64.93+/-10.59

Table 06. Regions (relatively to 5'/3' end of ORFs) characterized by different codons NFC distributions, estimated using different distance metrics, when considering an equal amount of RC samples in each window and for each codon (*E. coli:* 300 RC, *B. subtilis*: 200 RC, *M. musculus, C. elegans, S. cerevisiae:* 400 RC).

	Distance from 5' UTR [codons]			Distance from 3' UTR [codons]		
	JS	Hellinger	Energy	JS metric	Hellinger metric	Energy
E. coli	82	79	-	70	68	86
B. subtilis	71	80	69	72	69	63
M. musculus	83	83	71	74	74	68
C. elegans	82	82	81	71	73	75
S. cerevisiae	81	82	83	73	69	72

Table 07. For each organism the logL(best fit)/logL(log normal fit) and logL(best fit)/logL(normal fit) ratios are presented only for codons that their best mathematical fitting is not of a log-normal type (rounding at two digits precision). As seen from the results below, the ratio between log-likelihoods of the best mathematical fitting and log-normal fitting were bigger than 0.99; in comparison, the ratio between log-likelihoods of the best mathematical fitting and normal fitting were smaller than 0.78).

E. coli			*M. musculus*			*C. elegans*			*S. cerevisiae*		
Codon	logL(best fit)/ logL(log normal fit)	logL(best fit)/logL(normal fit)	Codon	logL(best fit)/ logL(log normal fit)	logL(best fit)/logL(normal fit)	Codon	logL(best fit)/ logL(log normal fit)	logL(best fit)/ logL(normal fit)	Codon	logL(best fit)/ logL(log normal fit)	logL(best fit)/ logL(normal fit)
AGA	0.99	0.6	AAA	1	0.72	AGG	0.99	0.59	ACA	1	0.43
CGG	1	0.59	AAG	1	0.78	CGA	1	0.58	ACC	0.99	0.49
			ACC	0.99	0.7	CGG	1	0.53	ACT	0.99	0.48
			ACG	0.99	0.72	CTA	0.99	0.57	AGC	1	0.5
			AGA	1	0.69	GCG	1	0.56	CAC	0.97	0.34
			AGC	1	0.64	TTA	1	0.62	CAT	1	0.45
			ATC	1	0.75				CCA	1	0.59
			CAC	1	0.7				CCG	1	0.56
			CAG	1	0.71				CGA	0.98	0.38
			CCC	0.99	0.61				CGC	1	0.56
			CCG	1	0.6				GCC	1	0.5
			CCT	1	0.52				GCG	1	0.47
			CGC	0.99	0.67				GCT	1	0.55
			CGT	1	0.72				GGT	1	0.59
			CTC	0.99	0.55				GTC	1	0.6
			CTG	0.99	0.67				TGG	0.99	0.49
			CTT	1	0.61						
			GAC	1	0.81						
			GAG	1	0.76						
			GAT	1	0.8						
			GCC	0.99	0.68						
			GCG	0.99	0.69						
			GGC	0.99	0.67						
			GGT	0.99	0.72						
			GTA	1	0.73						
			GTC	1	0.67						
			GTG	1	0.64						
			TAC	0.99	0.72						
			TCG	1	0.71						
			TGT	1	0.69						
			TTC	1	0.66						

Table 08. Comparing distances between codons NFC distributions of different organisms in the same (d_1) and different (d_2) domain of life for various distance measure types. The first column describes the compared organisms in the same domain *vs.* organisms from different domains (*E. coli* – EC*; B. subtilis* – BS*; M. musculus* – Mm*; C. elegans* – CE*; S. cerevisiae* – SC). The next three columns describe different calculations performed using the different distribution distance metrics: the first and second sub-columns show the median distance over all codon types for organisms in the same domain (d_1) and in the different domains (d_2). The third sub-column shows the p-value of the Wilcoxon test applied on the calculated distances d_1 and d_2. The results indicate that almost in all cases organisms from the same domain are significantly closer than organisms from different domains, regardless of the applied distribution distance metric function.

Compared distances	JS distance			Hellinger distance			Energy distance		
d_1 $vs.$ d_2	median d_1	median d_2	p-value	median d_1	median d_2	p-value	median d_1	median d_2	p-value
d_1(Mm,Ce) *vs.* d_2(Mm,Ec)	0.026	0.047	2.9e-19	0.13	0.18	1.7e-19	5.7e-05	8.3e-05	2.3e-09
d_1(Mm,Ce) *vs.* d_2(Mm,Bs)	0.026	0.11	1.7e-21	0.13	0.27	1.7e-21	5.7e-05	0.0002	3.8e-21
d_1(Mm,Sc) *vs.* d_2(Mm,Ec)	0.011	0.047	1.4e-16	0.089	0.18	1.1e-16	3.1e-05	8.3e-05	8.4e-09
d_1(Mm,Sc) *vs.* d_2(Mm,Bs)	0.011	0.11	4.2e-21	0.089	0.27	4.2e-21	3.1e-05	0.0002	3.0e-18
d_1(Ce,Mm) *vs.* d_2(Ce,Ec)	0.026	0.024	0.69	0.13	0.13	0.74	5.7e-05	2.5e-05	1.3e-09
d_1(Ce,Sc) *vs.* d_2(Ce,Bs)	0.0093	0.038	7.4e-19	0.08	0.16	1.10E-18	2.3e-05	7.3e-05	4.8e-12
d_1(Sc,Mm) *vs.* d_2(Sc,Ec)	0.011	0.032	7.3e-13	0.089	0.15	7.0e-13	3.1e-05	4.5e-05	8.7e-03
d_1(Sc,Ce) *vs.* d_2(Sc,Bs)	0.0093	0.061	2.2e-21	0.08	0.21	2.20e-21	2.3e-05	0.00011	2.4e-17
d_1(Ec,Bs) *vs.* d_2(Ec,Mm)	0.033	0.047	1.5e-10	0.15	0.18	1.0e-10	5.9e-05	8.3e-05	6.9e-06
d_1(Ec,Bs) *vs.* d_2(Ec,Ce)	0.033	0.024	5.6e-06	0.15	0.13	6.8e-06	5.9e-05	2.5e-05	2.0e-09
d_1(Ec,Bs) *vs.* d_2(Ec,Sc)	0.033	0.032	0.57	0.15	0.15	0.65	5.9e-05	4.5e-05	5.3e-03
d_1(Bs,Ec) *vs.* d_2(Bs,Mm)	0.033	0.11	1.7e-21	0.15	0.27	1.7e-21	5.9e-05	0.0002	2.4e-20
d_1(Bs,Ec) *vs.* d_2(Bs,Ce)	0.033	0.038	0.034	0.15	0.16	0.036	5.9e-05	7.3e-05	4.9e-02
d_1(Bs,Ec) *vs.* d_2(Bs,Sc)	0.033	0.061	3.4e-16	0.15	0.21	2.7e-16	5.9e-05	0.00011	1.9e-12
d_1(Ec,Bs) *vs.* d_2(Ec,Mm)	0.033	0.047	1.5e-10	0.15	0.18	1.0e-10	5.9e-05	8.3e-05	6.9e-06
Median value	0.0260	0.0470	3.4e-16	0.1300	0.1800	2.7e-16	5.7e-05	8.30e-05	2.0e-09

Table 09. Nominal decoding times used in the TASEP simulation

Codon	Nominal decoding times	Codon	Nominal decoding times
AAA	0.4149	GAA	0.2213
AAC	0.3017	GAC	0.2074
AAG	0.2004	GAG	0.4881
AAT	0.6873	GAT	0.4725
ACA	0.6637	GCA	0.5531
ACC	0.4191	GCC	0.4191
ACG	1.2766	GCG	1.7287
ACT	0.3017	GCT	0.3017
AGA	0.2766	GGA	1.1064
AGC	1.6595	GGC	0.2074
AGG	0.6858	GGG	1.1213
AGT	3.7803	GGT	0.4725
ATA	1.6585	GTA	1.1058
ATC	0.3204	GTC	0.3293
ATG	0.3017	GTG	1.1213
ATT	0.247	GTT	0.2371
CAA	0.3688	TAA	0
CAC	0.4149	TAC	0.4149
CAG	0.8554	TAG	0
CAT	0.9451	TAT	0.9451
CCA	0.3319	TCA	0.8295
CCC	2.3049	TCC	0.4191
CCG	1.0372	TCG	1.4557
CCT	1.6595	TCT	0.3017

CGA	0.5531	TGA	0
CGC	0.6586	TGC	0.8298
CGG	3.3191	TGG	0.5252
CGT	0.4742	TGT	1.8901
CTA	1.1064	TTA	0.4742
CTC	3.3191	TTC	0.3017
CTG	3.4574	TTG	0.2712
CTT	7.5606	TTT	0.6873

Table 10. Spearman correlation between tRNA copy numbers and various estimated scalar metrics representing basic NFC features

	corr(tRNA copy nums., mean)	corr(tRNA copy nums., median)	corr(tRNA copy nums., mode)	corr(tRNA copy nums., mean log-normal)	corr(tRNA copy nums., median log-normal)	corr(tRNA copy nums., skewness log-normal)
E. coli	r = -0.24, p = 0.14	r = -0.3, p = 0.068	r = -0.13, p = 0.43	r = -0.34, p = 0.033	r=-0.60, p = 5.7e-05	r = 0.36, p = 0.024
B. subtilis	r = 0.01, p = 0.94	r = -0.45, p = 0.0081	r = -0.71, p = 3.8e-06	r = -0.52, p = 0.0018	r=-0.72 p = 2.2e-06	r = 0.62 p = 0.00012
M. musculus	r = 0.01, p = 0.95	r = 0.04, p = 0.78	r = 0.17, p = 0.25	r = 0.04, p = 0.8	r=0.08 p = 0.59	r = -0.08, p = 0.56
C. elegans	r = 0.23, p = 0.12	r = 0.2, p = 0.18	r = 0.01, p = 0.92	r = 0.17, p = 0.25	r=-0.21 p = 0.16	r = 0.46, p = 0.0011
S. cerevisiae Brar	r = 0.19, p = 0.22	r = 0.2, p = 0.2	r = 0.14, p = 0.39	r = 0.18, p = 0.25	r=-0.05 p = 0.74	r = 0.37, p = 0.015
S. cerevisiae Ingolia	r = -0.02, p = 0.89	r = -0.13 p = 0.41	r = 0.07, p = 0.64	r = -0.23, p = 0.14	r = -0.59, p = 4.4e-05	r = 0.69, p = 3.6e-07

Table 11. Spearman correlation between codons tAI values and various estimated scalar metrics representing basic NFC features.

	corr (tAI, mean)	corr(tAI,median)	corr(tAI, mode)	corr(tAI, mean log-normal)	corr(tAI, median log-normal)	corr(tAI, skewness log-normal)
E. coli	r = -0.12, p = 0.38	r = -0.17, p = 0.18	r = -0.08, p = 0.53	r = -0.17, p = 0.19	r = -0.46, p = 0.00021	r = 0.36, p = 0.0047
B. subtilis	r = 0.13, p = 0.33	r= -0.08, p = 0.55	r = -0.46, p = 0.0001	r = -0.19, p = 0.15	r = -0.43, p = 0.00047	r = 0.42, p = 0.00072
M. musculus	r = -0.01, p = 0.92	r= 0.02, p = 0.86	r = 0.07, p = 0.58	r = 0.02, p = 0.86	r = 0.03, p = 0.83	r = 0.00, p = 0.99
C. elegans	r = 0.17, p = 0.18	r= 0.09, p = 0.47	r = -0.14, p = 0.28	r = 0.07, p = 0.57;	r = -0.32, p = 0.012	r = 0.54, p = 7.4e-06
S. cerevisiae Brar	r = 0.3, p = 0.019	r = 0.32, p = 0.012	r = 0.25, p = 0.054	r = 0.27, p = 0.033	r = 0.01, p = 0.91	r = 0.51, p = 3.3e-05
S. cerevisiae	r=0.02,	r=-0.05,	r=-0.10,	r = -0.19,	r = -0.54,	r = 0.71,

Ingolia	p = 0.88	p = 0.69	p = 0.43	p = 0.15	p = 7.2e-06	p = 1.9e-10

Table 12. Spearman correlation between tRNA copy numbers and various estimated scalar metrics representing basic NFC features, when considering an equal amount of RC for all codon types (*E. coli:* 2200 RC, *B. subtilis*: 1800 RC, *M. musculus, C. elegans, S. cerevisiae (Brar):* 5000 RC).

	corr(tRNA copy nums., mean)	corr(tRNA copy nums., median)	corr(tRNA copy nums., mode)	corr(tRNA copy nums., mean log-normal)	corr(tRNA copy nums., median log-normal)	corr(tRNA copy nums., skewness log-normal)
E. coli	r = -0.2,<br>p = 0.24	r = -0.31,<br>p = 0.064	r = -0.04,<br>p = 0.81	r = -0.29,<br>p = 0.12	r = -0.58,<br>p = 0.00064	r = 0.53,<br>p = 0.0022
B. subtilis	r = 0.03,<br>p = 0.88	r = -0.28,<br>p = 0.13	r = -0.67,<br>p = 0.00027	r=-0.34,<br>p = 0.093	r = -0.52,<br>p = 0.0083	r = 0.39,<br>p = 0.055
M. musculus	r = -0.03,<br>p = 0.83	r = -0.02,<br>p = 0.9	r = 0.22,<br>p = 0.16	r=0.01,<br>p = 0.96	r = 0.02,<br>p = 0.92,	r = 0.06,<br>p = 0.69
C. elegans	r = 0.32,<br>p = 0.074	r = 0.31,<br>p = 0.049	r = 0.21,<br>p = 0.19	r=0.32,<br>p = 0.043	r = -0.02,<br>p = 0.89	r = 0.47,<br>p = 0.0022
S. cerevisiae Brar	r = 0.13,<br>p = 0.45	r = 0.11,<br>p = 0.5	r = 0.07,<br>p = 0.69	r=0.09,<br>p = 0.59	r = -0.20,<br>p = 0.23	r = 0.38,<br>p = 0.017
S. cerevisiae Ingolia	Not enough read counts per codon for this test					

Table 13. Spearman correlation between codons tAI values and various estimated scalar metrics representing basic NFC features, when considering an equal amount of RC for all codon types (*E. coli:* 2200 RC, *B. subtilis*: 1800 RC, *M. musculus, C. elegans, S. cerevisiae:* 5000 RC).

	corr (tAI, mean)	corr(tAI,median)	corr(tAI, mode)	corr(tAI, mean log-normal)	corr(tAI, median log-normal)	corr(tAI, skewness log-normal)
E. coli	r= -0.05,<br>p = 0.7	r = -0.15,<br>p = 0.25	r = -0.08,<br>p = 0.56	r = -0.16,<br>p = 0.29,	r = -0.41,<br>p = 0.0034	r = 0.44,<br>p = 0.0015
B. subtilis	r = 0.15,<br>p = 0.27	r = -0.06,<br>p = 0.65	r = -0.4,<br>p = 0.0065	r = -0.09,<br>p = 0.55	r = -0.37,<br>p = 0.01	r=0.41,<br>p = 0.0044
M. musculus	r = -0.02,	r = -0.0,	r = 0.15,	r = 0.01,	r = -0.04,	r=0.13,

	p = 0.88	p = 0.99	p = 0.29	p = 0.96	p = 0.78	p = 0.35
C. elegans	r = 0.16, p = 0.25	r = 0.09, p = 0.49	r = -0.05, p = 0.72	r = 0.13, p = 0.36	r = -0.19, p = 0.17	r=0.48, p = 0.00028
S. cerevisiae Brar 2012	r = 0.18, p = 0.21	r = 0.18, p = 0.19	r = 0.03, p = 0.86	r = 0.11, p = 0.44	r = -0.18, p = 0.2	r=0.50, p = 0.00017
S. cerevisiae Ingolia	Not enough read counts per codon for this test					

Table 14: Spearman correlation between codon decoding time estimators of different experimental replicas, for the various analyzed organisms. For *C. elegans and M. musculus* only one experimental replica was made public.

Organism/estimator type	mean	median	mean of log-normal	median of log-normal	skewness of log-normal
E. coli	r = 0.99, p = 0	r = 0.97, p = 0	r = 0.98, p = 0	r = 0.94, p = 0	r = 0.96, p = 0
B. subtilis	r = 0.78, p = 0	r = 0.53, p = 1.5e-05	r = 0.58, p = 1.3e-06	r = 0.86, p = 0	r = 0.86, p = 0
S. cerevisiae Brar	r = 0.92, p = 0	r = 0.74, p = 0	r = 0.98, p = 0	r = 0.97, p = 0	r = 0.98, p = 0
S. cerevisiae Ingolia	r = 0.75, p = 0	r = 0.78, p = 1.3e-13	r = 0.87, p = 0	r = 0.84, p = 0	r = 0.81, p = 0

Supplementary references

1. Brar GA, Yassour M, Friedman N, Regev A, Ingolia NT, Weissman JS: **High-resolution view of the yeast meiotic program revealed by ribosome profiling**. *Science* 2012, **335**(6068):552-557.
2. Ingolia NT, Ghaemmaghami S, Newman JR, Weissman JS: **Genome-wide analysis in vivo of translation with nucleotide resolution using ribosome profiling**. *Science* 2009, **324**(5924):218-223.
3. Stadler M, Artiles K, Pak J, Fire A: **Contributions of mRNA abundance, ribosome loading, and post- or peri-translational effects to temporal repression of C. elegans heterochronic miRNA targets**. *Genome research* 2012.
4. Ingolia NT, Lareau LF, Weissman JS: **Ribosome profiling of mouse embryonic stem cells reveals the complexity and dynamics of mammalian proteomes**. *Cell* 2011, **147**(4):789-802.
5. Li GW, Oh E, Weissman JS: **The anti-Shine-Dalgarno sequence drives translational pausing and codon choice in bacteria**. *Nature* 2012, **484**(28):538-541.
6. Langmead B, Trapnell C, Pop M, Salzberg SL: **Ultrafast and memory-efficient alignment of short DNA sequences to the human genome**. *Genome Biol* 2009, **10**(3):R25.
7. Qian W, Yang JR, Pearson NM, Maclean C, Zhang J: **Balanced codon usage optimizes eukaryotic translational efficiency**. *PLoS genetics* 2012, **8**(3):e1002603.
8. Charneski CA, Hurst LD: **Positively charged residues are the major determinants of ribosomal velocity**. *PLoS biology* 2013, **11**(3):e1001508.
9. Zinshteyn B, Gilbert WV: **Loss of a conserved tRNA anticodon modification perturbs cellular signaling**. *PLoS genetics* 2013, **9**(8):e1003675.
10. Dana A, Tuller T: **Determinants of translation elongation speed and ribosomal profiling biases in mouse embryonic stem cells**. *PLoS computational biology* 2012, **8**(11):e1002755.
11. Hsu F, Kent WJ, Clawson H, Kuhn RM, Diekhans M, Haussler D: **The UCSC Known Genes**. *Bioinformatics* 2006, **22**(9):1036-1046.
12. Ingolia NT, Brar GA, Rouskin S, McGeachy AM, Weissman JS: **The ribosome profiling strategy for monitoring translation in vivo by deep sequencing of ribosome-protected mRNA fragments**. *Nature protocols* 2012, **7**(8):1534-1550.
13. Kullback S, Leibler RA: **On information and sufficiency**. *The Annals of Mathematical Statistics* 1951, **22**(1):79-86.
14. dos Reis M, Savva R, Wernisch L: **Solving the riddle of codon usage preferences: a test for translational selection**. *Nucleic Acids Res* 2004, **32**(17):5036-5044.
15. Shaw LB, Zia RK, Lee KH: **Totally asymmetric exclusion process with extended objects: a model for protein synthesis**. *Phys Rev E Stat Nonlin Soft Matter Phys* 2003, **68**(2 Pt 1):021910.
16. Tuller T, Veksler-Lublinsky I, Gazit N, Kupiec M, Ruppin E, Ziv-Ukelson M: **Composite effects of gene determinants on the translation speed and density of ribosomes**. *Genome Biol* 2011, **12**(11):R110.
17. Reuveni S, Meilijson I, Kupiec M, Ruppin E, Tuller T: **Genome-scale analysis of translation elongation with a ribosome flow model**. *PLoS computational biology* 2011, **7**(9):e1002127.
18. Dana A, Tuller T: **The effect of tRNA levels on decoding times of mRNA codons**. *Under review* 2014.
